# Dynamic modulations of sphingolipids and glycerophospholipids in COVID‐19

**DOI:** 10.1002/ctm2.1069

**Published:** 2022-10-10

**Authors:** Makoto Kurano, Koh Okamoto, Daisuke Jubishi, Hideki Hashimoto, Eri Sakai, Daisuke Saigusa, Kuniyuki Kano, Junken Aoki, Sohei Harada, Shu Okugawa, Kent Doi, Kyoji Moriya, Yutaka Yatomi

**Affiliations:** ^1^ Department of Clinical Laboratory Medicine Graduate School of Medicine The University of Tokyo Tokyo Japan; ^2^ Department of Infectious Diseases Graduate School of Medicine The University of Tokyo Tokyo Japan; ^3^ Laboratory of Biomedical and Analytical Sciences Faculty of Pharma‐Science Teikyo University Tokyo Japan; ^4^ Department of Health Chemistry Graduate School of Pharmaceutical Sciences The University of Tokyo Tokyo Japan; ^5^ Department of Infection Control and Prevention The University of Tokyo Tokyo Japan; ^6^ Department of Emergency and Critical Care Medicine The University of Tokyo Hospital, Tokyo, Japan

**Keywords:** COVID‐19, glycerophospholipids, lipidomics, severity, sphingolipids

## Abstract

**Background:**

A heterogeneous clinical phenotype is a characteristic of coronavirus disease 2019 (COVID‐19). Therefore, investigating biomarkers associated with disease severity is important for understanding the mechanisms responsible for this heterogeneity and for developing novel agents to prevent critical conditions. This study aimed to elucidate the modulations of sphingolipids and glycerophospholipids, which have been shown to possess potent biological properties.

**Methods:**

We measured the serum sphingolipid and glycerophospholipid levels in a total of 887 samples from 215 COVID‐19 subjects, plus 115 control subjects without infectious diseases and 109 subjects with infectious diseases other than COVID‐19.

**Results:**

We observed the dynamic modulations of sphingolipids and glycerophospholipids in the serum of COVID‐19 subjects, depending on the time course and severity. The elevation of C16:0 ceramide and lysophosphatidylinositol and decreases in C18:1 ceramide, dihydrosphingosine, lysophosphatidylglycerol, phosphatidylglycerol and phosphatidylinositol were specific to COVID‐19. Regarding the association with maximum severity, phosphatidylinositol and phosphatidylcholine species with long unsaturated acyl chains were negatively associated, while lysophosphatidylethanolamine and phosphatidylethanolamine were positively associated with maximum severity during the early phase. Lysophosphatidylcholine and phosphatidylcholine had strong negative correlations with CRP, while phosphatidylethanolamine had strong positive ones. C16:0 ceramide, lysophosphatidylcholine, phosphatidylcholine and phosphatidylethanolamine species with long unsaturated acyl chains had negative correlations with D‐dimer, while phosphatidylethanolamine species with short acyl chains and phosphatidylinositol had positive ones. Several species of phosphatidylcholine, phosphatidylethanolamine and sphingomyelin might serve as better biomarkers for predicting severe COVID‐19 during the early phase than CRP and D‐dimer. Compared with the lipid modulations seen in mice treated with lipopolysaccharide, tissue factor, or histone, the lipid modulations observed in severe COVID‐19 were most akin to those in mice administered lipopolysaccharide.

**Conclusion:**

A better understanding of the disturbances in sphingolipids and glycerophospholipids observed in this study will prompt further investigation to develop laboratory testing for predicting maximum severity and/or novel agents to suppress the aggravation of COVID‐19.

## Graphical abstract

- The dynamic modulations of sphingolipids and glycerophospholipids were observed in the serum of COVID‐19 subjects, depending on the time course and severity.
- Several species of phosphatidylcholine, phosphatidylethanolamine, and sphingomyelin might serve as better biomarkers for predicting severe COVID‐19 during the early phase than CRP and D‐dimer.
- These results may help researchers to develop laboratory testing and/or novel agents for COVID‐19.

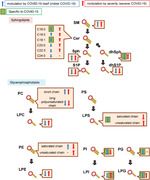

## 1 INTRODUCTION

With the global coronavirus disease 2019 (COVID‐19) pandemic, caused by severe acute respiratory syndrome coronavirus 2 (SARS‐CoV‐2), still ongoing, the investigation of biomarkers associated with COVID‐19 remains an important task. Since the heterogeneity of clinical phenotypes of COVID‐19 has made this disease difficult to deal with, the identification of biomarkers of COVID‐19 severity is needed to understand the mechanisms responsible for this heterogeneity and to develop novel agents capable of preventing severe conditions. At present, biological responses, such as immunological overreactions, as well as viral factors, are thought to be involved in the severity of COVID‐19. Among the biological responses, a series of elegant studies revealed that bioactive lipids are generated during inflammatory and immune responses and contribute to their regulation.^1^, ^2^ In the present study, we focused on sphingolipids and glycerophospholipids.

Among sphingolipids, the bioactivities of sphingosine 1‐phosphate (S1P) and ceramides have been widely studied. Regarding organ injuries and inflammation, S1P possesses potent anti‐apoptotic and pro‐survival properties^3^ and generally suppresses inflammation,^4^ while ceramides facilitate apoptosis^5^ and inflammation.^6^ At present, five kinds of S1P receptors, S1P1–S1P5, have been identified, and S1P is produced from sphingosine (Sph) by S1P kinases.^3^ Ceramides are derived from sphingomyelin (SM) and can be converted into Sph. In addition to S1P, dihydrosphingosine 1‐phosphate (dhS1P) is known as another analogue for S1P receptors. DhS1P is produced from dihydrosphingosine (dhSph), and dhSph can be processed into ceramides via dihydroceramides.^7^

Among glycerophospholipids, receptors for lysophosphatidic acids (LPA), lysophosphatidylserine (LPS), lysophosphatidylinositol (LPI) and lysophosphatidylglycerol (LPG) have been identified.^8^ LPA is produced mainly from lysophosphatidylcholine (LPC) by autotaxin. The roles of LPA in inflammation and immune response depend on six kinds of LPA receptors, LPA1–LPA6. LPA1, LPA2 and LPA6 might aggravate the immune response, whereas LPA3–5 might attenuate it.^9^, ^10^, ^11^ LPC is produced from phosphatidylcholine (PC). Recent studies have also suggested the possible involvement of LPS in the immune response. Three kinds of LPS receptors, namely GPR34, P2Y10 and GPR174, have been identified. LPS suppresses the activation of T lymphocytes through GPR174,^12^ while LPS facilitates the migration of CD4 T cells through P2Y10.^13^ LPI and LPG act on GPR55, and this axis has been considered as a pro‐inflammatory pathway.^14^ LPI and LPG are produced from phosphatidylinositol (PI) and phosphatidylglycerol (PG), respectively. Lysophosphatidylethanolamine (LPE) and its precursor phosphatidylethanolamine (PE) have been shown to be involved in the immune response.^15^

Until now, several mass spectrometry studies have performed lipidomics analyses of samples collected from COVID‐19 subjects. However, few studies included a large number (over 200) of subjects, and none of these studies investigated the modulations of sphingolipids and glycerophospholipids longitudinally in detail. Since the clinical conditions of patients with COVID‐19 can change dramatically on a daily basis, the investigation of serial changes in lipid levels is important. Therefore, we measured the serum sphingolipid and glycerophospholipid levels in a total of 887 samples from 215 COVID‐19 subjects together with 115 control subjects without infectious diseases and 109 subjects with infectious diseases other than COVID‐19.

## 2 RESULTS

### 2.1 Glycerolysophospholipids other than LPA, diacylphospholipids other than PS, and sphingolipids other than S1P and dhS1P were reasonably evaluated by measuring their serum concentrations

Since the sampling of plasma samples under strict conditions, as performed in a previous study,^16^ was difficult because of biosafety concerns at our hospital, we first determined what types of lipids could be reasonably evaluated using serum samples. As shown in Figure S1, the LPA, S1P, dhS1P and PS levels were quite different between serum samples and plasma samples collected under strict conditions. Although a significant difference was observed for LPS, LPI and C16:0 ceramide, the differences were within 10%, suggesting that the modulations of these lipids could be evaluated using serum samples. Regarding the other lipids, no significant differences were observed. Considering these results, we deemed that the serum concentrations of the measured lipids, excluding LPA, S1P, dhS1P and PS, suitably reflected those in vivo.

### 2.2 Sphingomyelin, sphingosine and ceramides were modulated in time‐course and severity‐dependent manners in COVID‐19 subjects

**FIGURE 1:**
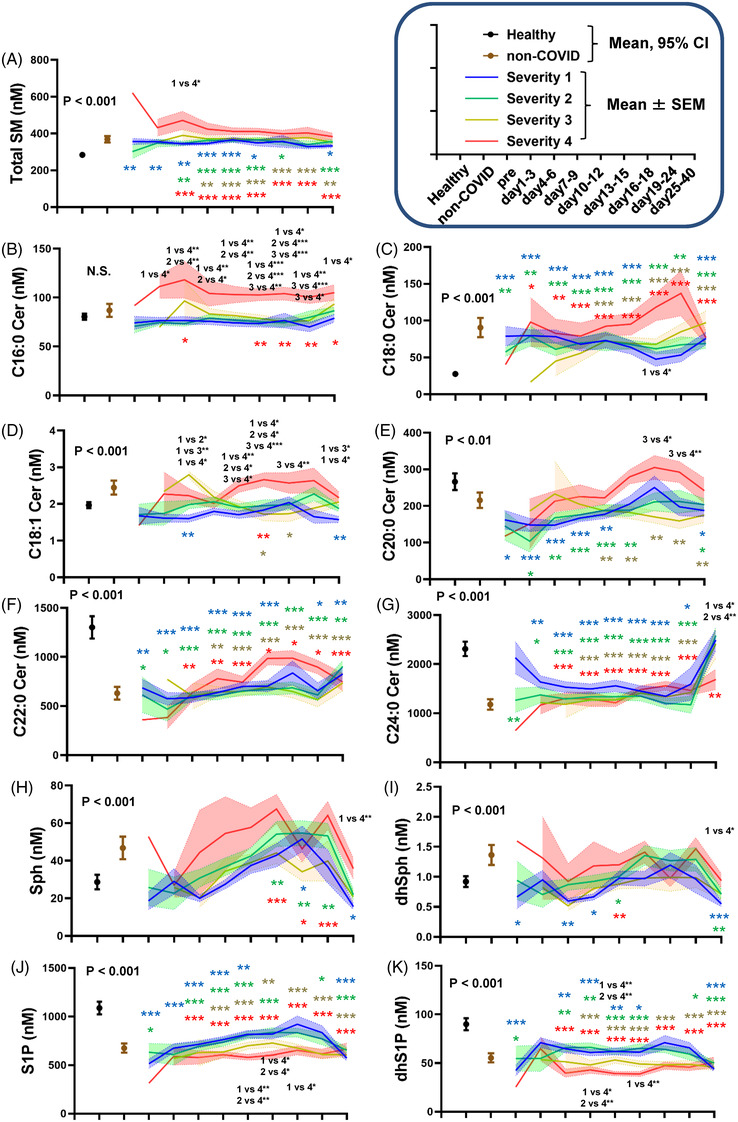
Modulations of sphingolipids during the time course of COVID‐19. Serum sphingolipid levels were measured longitudinally in symptomatic COVID‐19 subjects (*n* = 203). The significance of the Steel–Dwass test following the Kruskal–Wallis test among healthy subjects and COVID‐19 subjects with a specific maximum severity is shown as *p < .05, **p < .01, or ***p < .001 for the comparison with healthy subjects or between COVID‐19 subjects with a specific maximum severity. Blue, green, yellow and red stars represent the difference between healthy subjects and, maximum severity 1, maximum severity 2, maximum severity 3, or maximum severity 4. The difference among COVID‐19 subjects with a specific maximum severity is described in black. The difference between healthy subjects and subjects with non‐COVID‐19 infectious diseases was evaluated with the Mann–Whitney U test. The ranges in healthy subjects (*n* = 115) and those in subjects with non‐COVID infectious diseases (*n* = 109) are shown as the 95% confidence interval (CI); the ranges and those in COVID‐19 subjects with specific maximum severity defined in the Methods section are shown as mean ± SEM.

Figure 1 shows the time course for the sphingolipid modulations. The serum total SM levels increased in the COVID‐19 subjects as well as the subjects with non‐COVID‐19 infectious diseases (Figure 1A). The extent of these increases was greater in subjects with severe COVID‐19. The SM levels were higher even before the onset of COVID‐19 and in asymptomatic COVID‐19 subjects (Figure S2G). The SM levels were maintained at higher levels throughout the monitored time course, although the extent of the SM increase was particularly large from days 1 to 9 in maximum severity group 4.

Ceramide modulations seemed to depend on the ceramide species and the maximum severity of COVID‐19. The C16:0 ceramide levels increased only in maximum severity group 4 (Figure 1B). The C18:0 ceramide levels were higher in the COVID‐19 subjects, especially in maximum severity group 4 (Figure 1C). The C18:1 ceramide level decreased in maximum severity groups 1 and 3, while they increased in maximum severity group 4 (Figure 1D). The C20:0 and C22:0 ceramide levels decreased in the COVID‐19 subjects, while the extent of the decrease was rather small in maximum severity group 4 (Figure 1E,F). The C24:0 ceramide levels were lower in the COVID‐19 subjects and might have been independent of disease severity (Figure 1G). Significant modulations of the C18:0, C20:0, C22:0 and C24:0 ceramide levels were observed even before the onset of COVID‐19, while modulations of the C18:0, C22:0 and C24:0 ceramide levels were observed in asymptomatic COVID‐19 subjects (Figure S2A–F).

The Sph levels seemed to increase, especially in maximum severity group 4, while those of dhSph decreased in maximum severity group 1 (Figure 1H,I). On days 25–40, they were significantly lower in COVID‐19 subjects with a milder maximum severity. The dhSph levels were lower in maximum severity group 1 even before disease onset. Regarding S1P and dhS1P, they were lower in the COVID‐19 subjects, with larger extents seen in the higher maximum severity groups (Figure 1J,K). Significant reductions in S1P and dhS1P were also observed before disease onset and/or in asymptomatic subjects (Figure S2J).

For the subjects with non‐COVID‐19 infectious diseases, most of the monitored sphingolipids were modulated in the same directions as those seen in subjects with COVID‐19. However, the increase in C16:0 ceramide in maximum severity group 4 and the decreases in C18:1 ceramide and dhSph in the milder maximum severity groups were unique to COVID‐19.

### 2.3 Serum PC and PE increased and PG decreased in COVID‐19 subjects, while serum LPC, LPE and LPI decreased in COVID‐19 subjects with mild disease but increased in COVID‐19 subjects with severe disease

**FIGURE 2:**
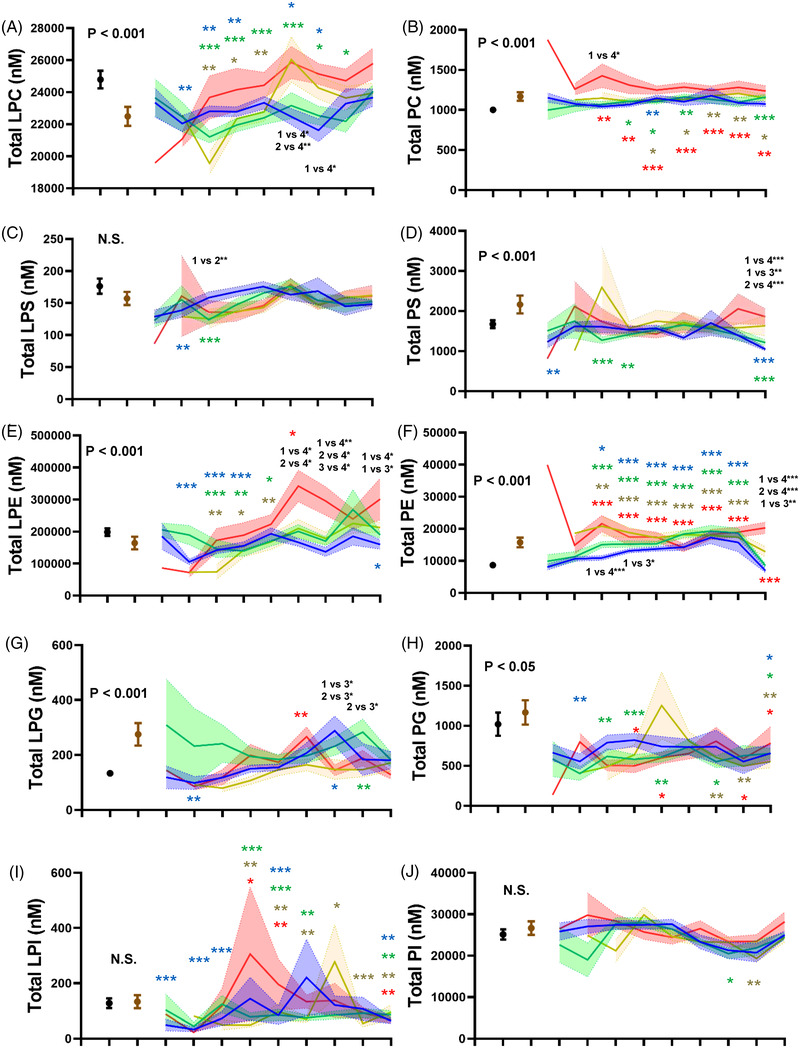
The modulations of glycerophospholipids during the time course of COVID‐19. The total levels of serum glycerophospholipids were measured longitudinally in symptomatic COVID‐19 subjects (*n* = 203). The significance of the Steel–Dwass test following the Kruskal–Wallis test among healthy subjects and COVID‐19 with specific maximum severity is shown as *p < .05, **p < .01, or ***p < .001 for the comparison with healthy subjects or between subjects with specific maximum severity. Blue, green, yellow and red stars represent the difference between healthy subjects and, maximum severity 1, maximum severity 2, maximum severity 3, or maximum severity 4. The difference among COVID‐19 subjects with a specific maximum severity is described in black. The difference between healthy subjects and subjects with non‐COVID‐19 infectious diseases was evaluated with the Mann–Whitney U test. The ranges in healthy subjects (*n* = 115) and those with non‐COVID infectious diseases (*n* = 109) are shown as 95 % confidence interval (CI) the ranges in COVID‐19 subjects with a specific maximum severity defined in the Methods section are shown as the mean ± SEM.

Figure 2 shows an overview of the modulations of all the serum glycerophospholipids. The total LPC levels decreased in maximum severity groups 1–3 but recovered by days 25–40, while the total LPC levels were not modulated in maximum severity group 4 (Figure 2A). The total PC levels increased in all the maximum severity groups, especially maximum severity group 4, on days 4–6 (Figure 2B). Decreased total LPS levels were observed in maximum severity groups 1 and 2 during the early phase (Figure 2C). The serum total PS levels were significantly lower in maximum severity groups 1 and 2 at several time points (Figure 2D). The serum LPE levels were modulated in different directions between maximum severity groups 1–3 and group 4. The serum total LPE levels decreased on days 1–12 in maximum severity groups 1–3, whereas they increased on days 13–15 in maximum severity group 4 (Figure 2E). The serum total PE levels increased in all the maximum severity groups (Figure 2F). The serum total LPG levels decreased during the early phase (days 1–3) in maximum severity group 1, while they increased significantly during the late phase (Figure 2G). The total PG levels remained at lower levels throughout the monitored time course (Figure 2H). The serum LPI levels were modulated in different directions, depending on the maximum severity of COVID‐19 and the time course. The serum LPI levels were lower during the early phase of the disease from symptom onset until days 25–40 in maximum severity group 1, while the serum total LPI levels increased on days 7–12 in maximum severity group 4 (Figure 2I). The serum total PI levels were not modulated dramatically (Figure 2J). In asymptomatic subjects, the total LPC and LPI levels decreased significantly, while the total PC levels increased significantly, compared with the levels in healthy subjects (Figure S2L–U).

### 2.4 Serum sphingolipid and glycerophospholipid levels contributed to the differentiation of COVID‐19 subjects from healthy subjects and subjects with non‐ COVID‐19 infectious diseases, depending on the time course and lipid species

**FIGURE 3:**
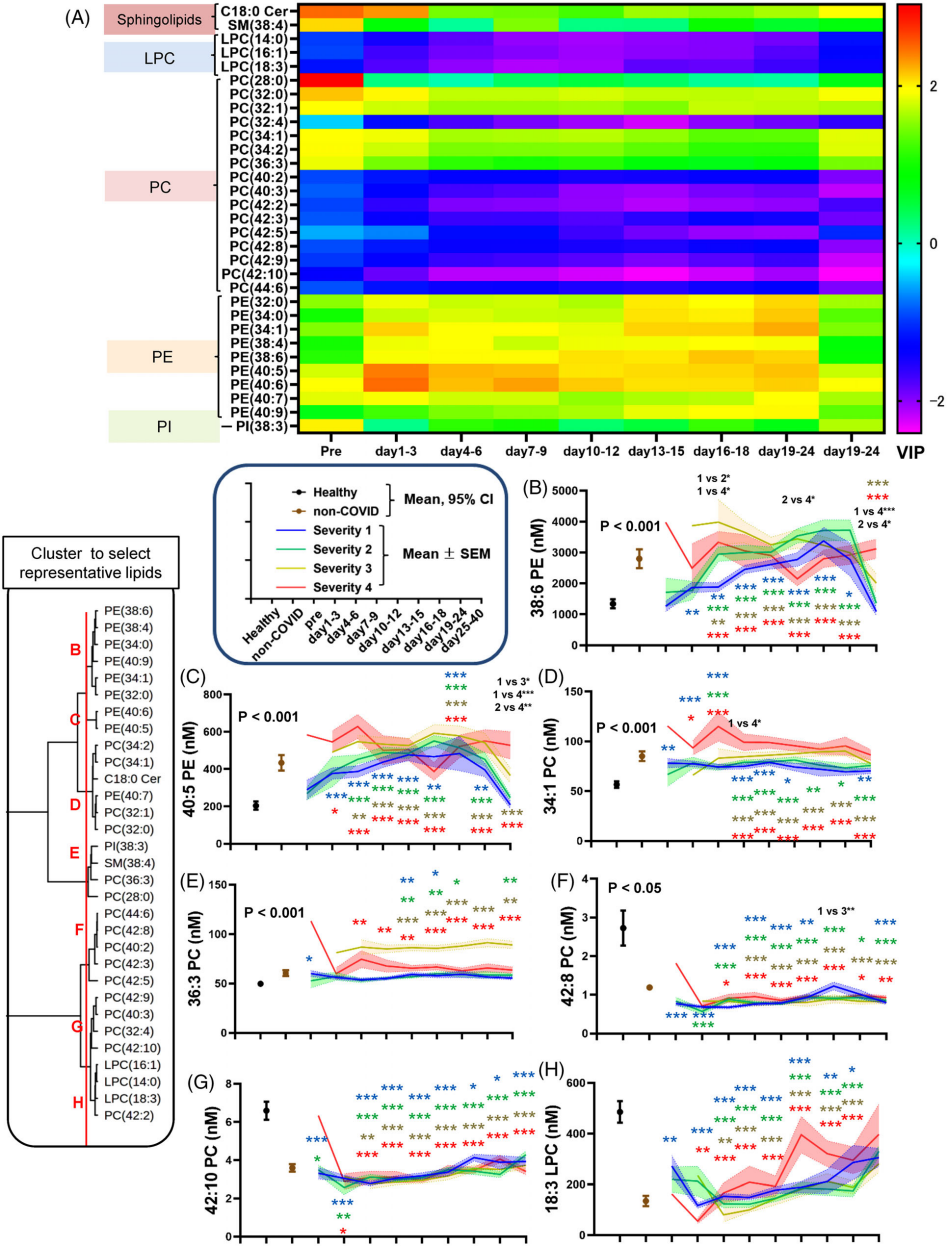
Important lipids for differentiating COVID‐19 subjects from healthy subjects in an OPLS‐DA. An OPLS‐DA using the measured lipid levels (excluding PS, S1P and dhS1P) and the clinical phenotypes was performed to investigate significant lipid modulations characteristic of COVID‐19 subjects, compared with healthy subjects. (A) Time courses of the VIP scores of the T scores for the top 10 most important lipids for differentiating both groups at any time point, shown as a heat map. (B–H) Time courses of lipids that were representative of lipid clusters are shown in a manner similar to that used in Figures 1 and 2.

Although a principal component analysis (PCA) could not completely discriminate COVID‐19 subjects from healthy subjects, their distributions did not thoroughly overlap each other (Figure S3). Therefore, to understand the lipid modulations characteristic of COVID‐19 subjects better, compared with healthy subjects, we performed an orthogonal projection to latent structure discriminant analysis (OPLS‐DA) using lipid levels (excluding PS, S1P and dhS1P). The detailed results are shown in Figures S4A–F, S5A–F, and S6A–F. To understand the results and the influence of the time course, the time courses of the variable importance in projection (VIP) scores of the T scores for the top 10 most important lipids for differentiating both groups at any time point were plotted as a heat map (Figure 3A). To view the time courses of representative lipids, we selected lipids using cluster analyses based on the time courses of the VIP scores in the OPLS‐DA. The time courses of the lipids that were selected as being representative of lipid clusters are shown in Figure 3B–H.

**FIGURE 4:**
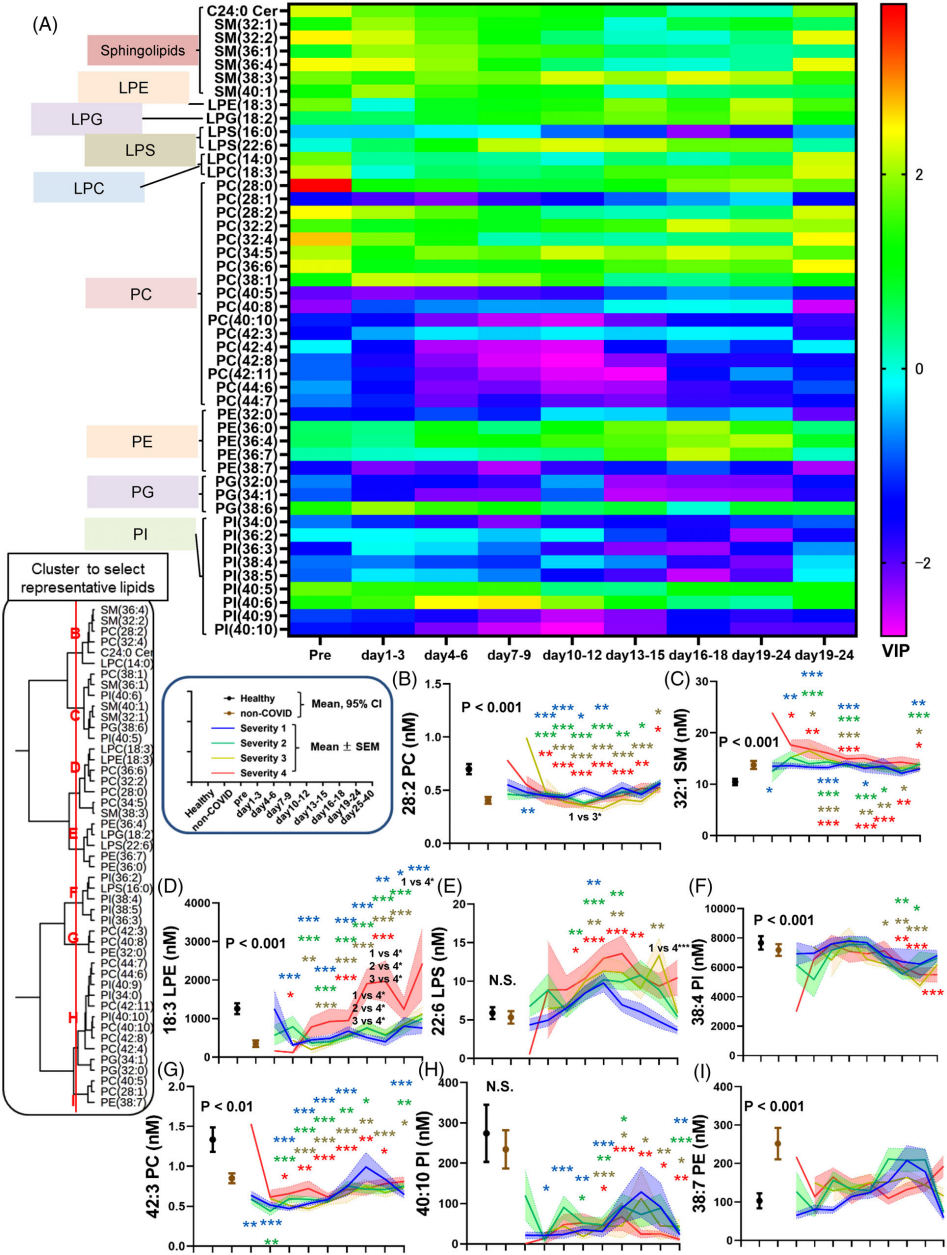
Important lipids for differentiating COVID‐19 subjects from subjects with non‐COVID‐19 infectious diseases in an OPLS‐DA. An OPLS‐DA using the measured lipid levels (excluding PS, S1P and dhS1P) and the clinical phenotypes was performed to investigate significant lipid modulations characteristic of COVID‐19 subjects, compared with subjects with non‐COVID‐19 infectious diseases. (A) Time courses of the VIP scores of the T scores for the top 10 most important lipids for differentiating both groups at any time point, shown as a heat‐map. (B–I) Time courses of lipids that were representative of lipid clusters are shown in a manner similar to that used in Figures 1 and 2.

The directions of the modulations of many sphingolipids and glycerophospholipids in the COVID‐19 subjects resembled those in subjects with non‐COVID‐19 infectious diseases (Figures 1 and 2). When we perform a PCA, although not completely separated, the distribution of the COVID‐19 subjects and that of the subjects with non‐COVID‐19 infectious diseases could be partially discriminated (Figure S7). Therefore, we next performed an OPLS‐DA to investigate significant lipid modulations characteristic of COVID‐19 subjects, compared to subjects with non‐COVID‐19 infectious diseases. The detailed results are shown in Figures S4G–L, S5G–L, and S6G–L. To understand the results and the influence of the time courses, the VIP scores of the lipids are shown in Figure 4A. Figure 4B–I shows representative lipids selected by cluster analyses.

### 2.5 PI and PC, especially PC species with long unsaturated acyl chains, were negatively and PE, except for 40:1 PE, was positively associated with maximum severity during the early phase

**FIGURE 5:**
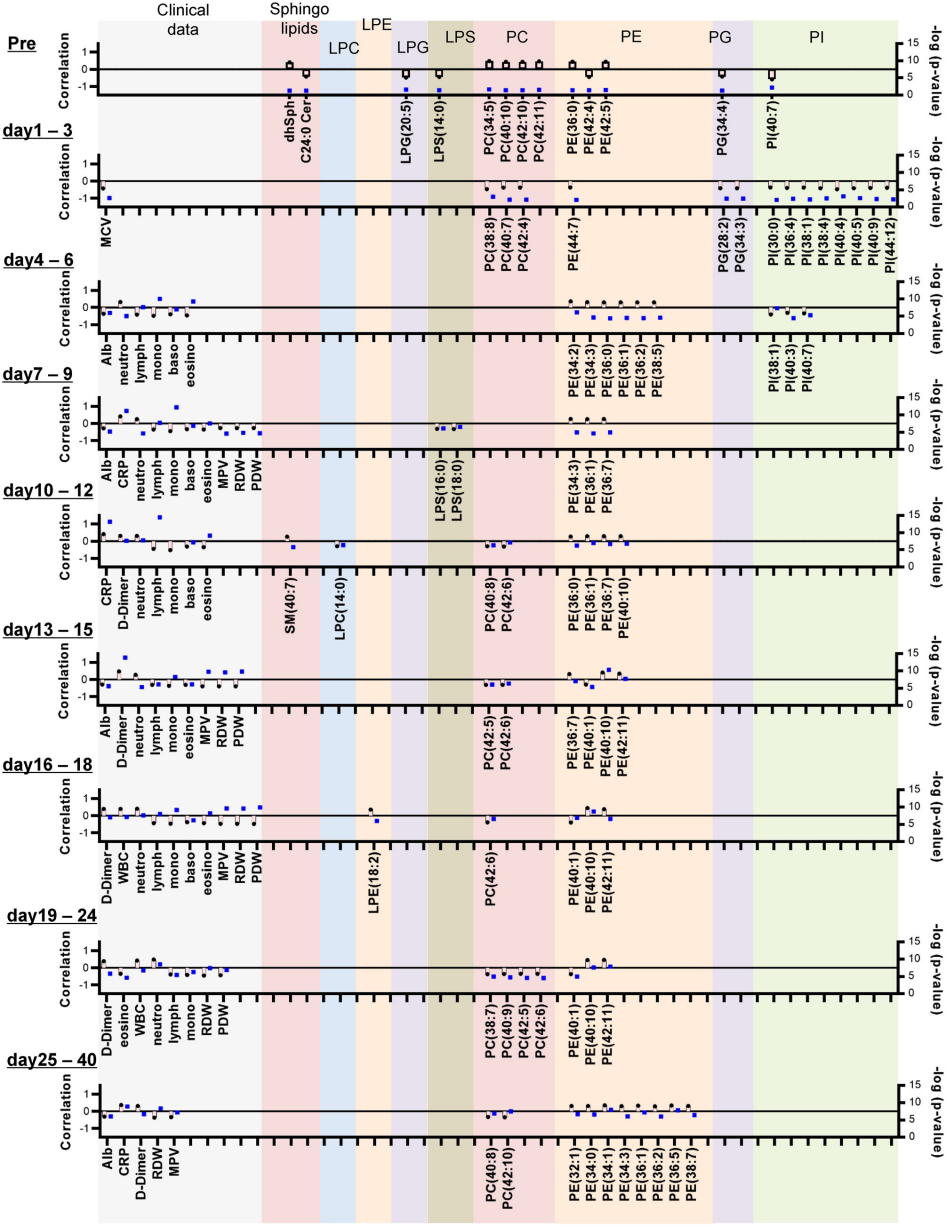
Associations of monitored lipids with the maximum severity of COVID‐19. Kendall rank correlation analyses were performed between lipids or clinical parameters and the maximum severity of COVID‐19, considering age, sex and the presence of diabetes, hypertension and current smoking as covariates of interest. Time courses of the correlation coefficients and *p* values (shown as ‐log_10_ [*p* value]) of the lipids and clinical parameters with the top 15 *p* values at a specific time point

Next, to investigate the association of sphingolipids and glycerophospholipids with the maximum severity of COVID‐19 at each time point, we performed correlation analyses using age, sex and the presence of diabetes, hypertension and current smoking as covariates of interest. Figure 5 shows the correlation coefficients and *p* values of the lipids and clinical parameters with the top 15 *p* values at each time point. During the early phases of days 1–3 and days 4–6, several PI species had negative correlations with maximum severity; later, most PE species had positive correlations. Notably, 40:1PE had a unique positive correlation with maximum severity, the time course of which is shown in Figure S8A. From days 10–12, PC species with long unsaturated acyl chains had negative correlations with relatively low *p* values. The *p* values of the PE and PC species were as low as those for the clinical laboratory data. Interestingly, several PC species with long unsaturated acyl chains had positive correlations with maximum severity even before symptom onset, although the *p* values were relatively high. The time courses of the representative lipids are shown in Figure S8B–G.

### 2.6 Sphingolipids and glycerophospholipids were significantly correlated with CRP and D‐dimer depending on lipid species and sampling point

**FIGURE 6:**
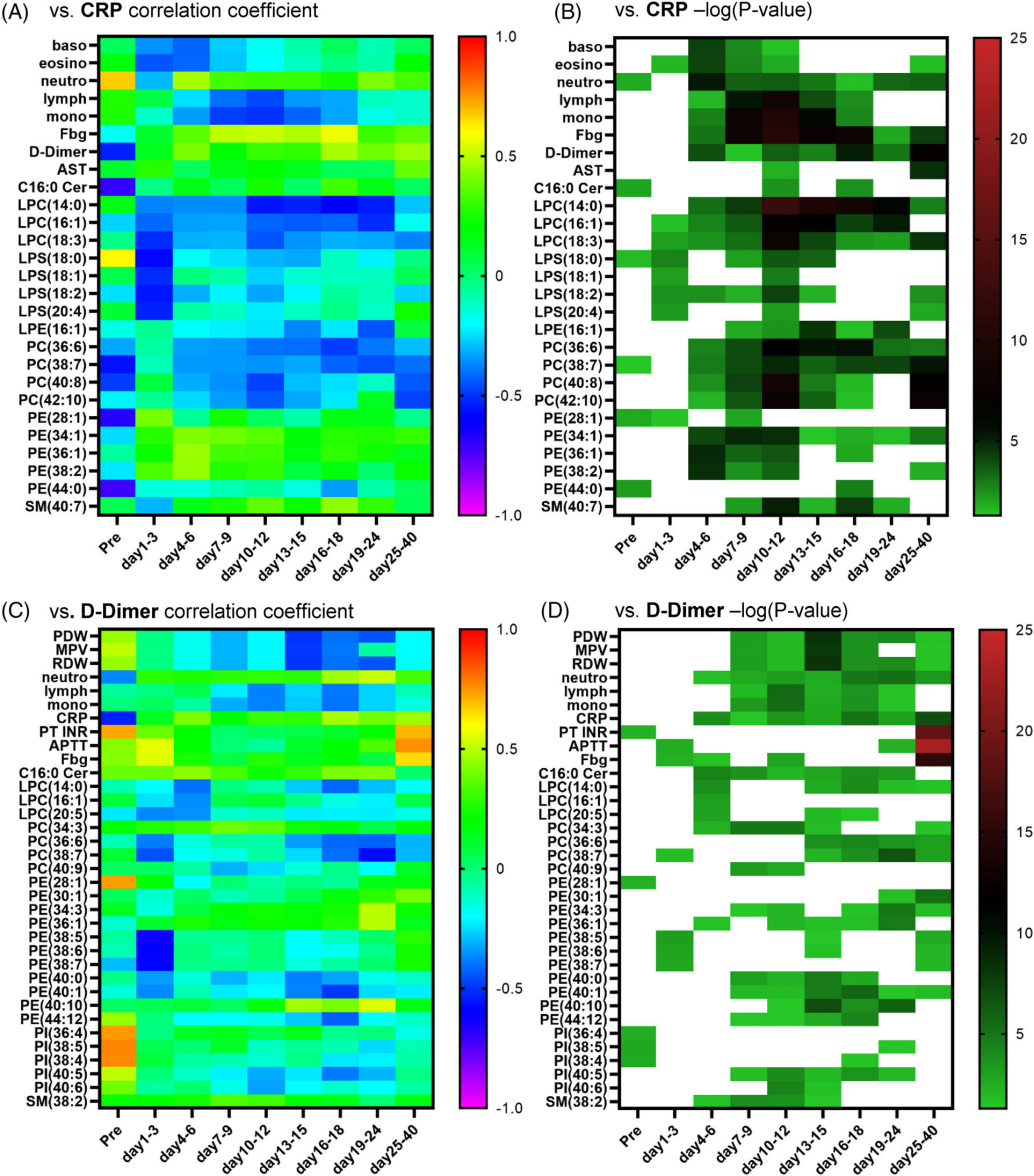
Time courses for the correlations of the monitored lipids with CRP and D‐dimer. Spearman rank correlation analyses were used to compare the lipids or clinical parameters and CRP (A,B) or D‐dimer (C,D) at specific time points. The time courses of the correlation coefficients (A,C) and the *p* values (B,D, shown as ‐log_10_ [*p* value]) of the lipids and clinical parameters with the top 10 *p* values at any time point are shown as a heat‐map. The correlation coefficients and *p* values of non‐significant results are shown as blanks.

Figure 6 shows the time courses for the correlation coefficients with the CRP and D‐dimer levels of the top 10 lipids with the lowest *p* values at any time point as a heat map. For the associations with CRP (Figure 6A,B), several LPC species and PC species had rather strong negative correlations throughout the time course, while several PE species had rather strong positive correlations. We also observed rather strong negative correlations of 18:0 LPS, 18:1 LPS, 18:2 LPS and 16:1 LPE and positive correlations of 40:7 SM with the CRP levels. The time courses of the representative lipids are shown in Figure S9. For the associations with D‐dimer (Figure 6C,D), C16:0 ceramide and 38:2 SM had positive correlations. Among LPC species, 14:0 LPC, 16:1 LPC and 20:5 LPC had rather strong negative correlations. Among PC species, the association with D‐dimer depended on the PC species: 40:7 PC had negative correlations, whereas 34:3 PC had positive correlations. Meanwhile, 36:6 PC and 38:7 PC had rather strong negative correlations after days 13–15. Among PE and PI species, the associations with D‐dimer depended not only on their species, but also on the sampling points. The time courses for the representative lipids are shown in Figure S10.

### 2.7 Several different correlations between lipids and clinical parameters, especially D‐dimer levels, were observed between COVID‐19 subjects and subjects with non‐COVID‐19 infectious diseases

**FIGURE 7:**
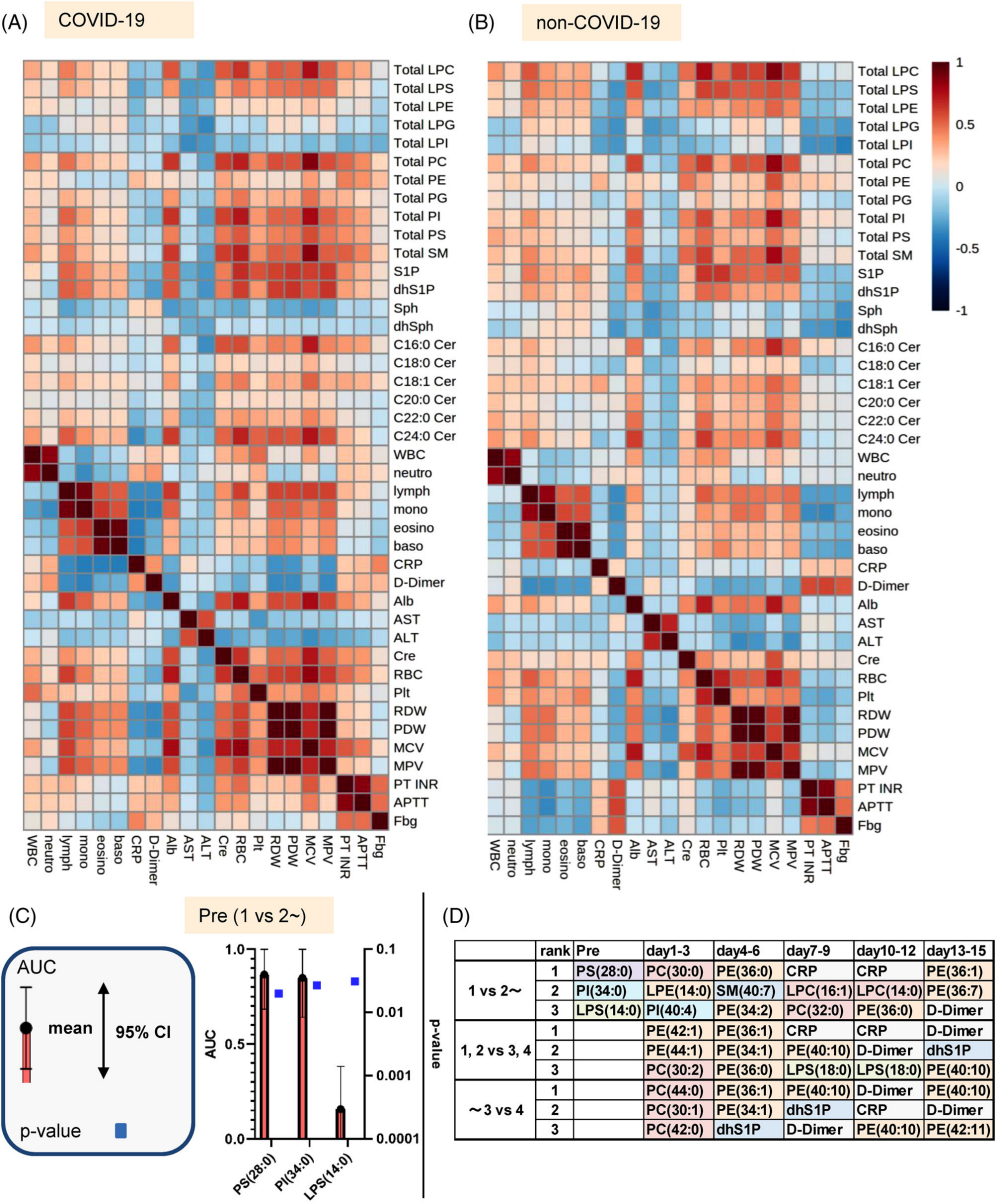
Correlations between the monitored lipids and clinical parameters in COVID‐19 subjects and subjects with non‐COVID‐19 infectious diseases and ROC analyses for the prediction of maximum severity. (A,B) Spearman rank correlation analyses for the lipids and clinical parameters in all the analysed samples obtained from symptomatic COVID‐19 subjects (A) and subjects with non‐COVID‐19 infectious diseases (B). (C,D) Area under curve (AUC) of the ROC analyses for differentiating maximum severity groups 2–4 from maximum severity group 1 before symptom onset: (C) AUC before symptom onset. (D) Time course of the parameters with the top three lowest *p* values at a specific time point

When we investigated the correlations with other clinical parameters in all the samples collected from symptomatic COVID‐19 subjects and compared the results with those for the subjects with non‐COVID‐19 infectious diseases, some differences were observed between the two groups in terms of the directions of the correlations (Figure 7A,B). The directions of the correlations of the total SM levels with the clinical parameters were the same between the two groups. Among the ceramides, C22:0 ceramide had significant positive correlations with the CRP and D‐dimer levels in the COVID‐19 subjects; C24:0 ceramide had significant positive correlations with PT‐INR and APTT and a negative correlation with the fibrinogen level, while the direction of the correlations was negative in the subjects with non‐COVID‐19 infectious diseases. The correlations of Sph or dhSph with the complete blood counts and the D‐dimer level were also in different directions in the two groups. In COVID‐19 subjects, significant positive correlations with the D‐dimer level were observed, whereas significant negative correlations were observed in subjects with non‐COVID‐19 infectious diseases. Regarding glycerophospholipids, some complete blood count parameters had different correlations with LPE, LPI and PE. The total LPC, LPS, LPE, PG and PI levels also had different correlations with some coagulation test parameters. In particular, LPE had a positive correlation with the D‐dimer level in COVID‐19 subjects, while it had a negative correlation in subjects with non‐COVID‐19 infectious diseases.

### 2.8 ROC analyses revealed the possibility that several species of PC, PE and SM might serve as better biomarkers for differentiating the maximum severity of COVID‐19 during the early phase (days 1–6) than CRP and D‐dimer

To compare the abilities of the measured lipids and CRP or D‐dimer levels to predict the maximum severity of COVID‐19, we performed ROC analyses. The top 20 parameters with the lowest *p* values are shown in Figure 7C,D and Figures S11–S13. Although the *p* values were rather high because of the small number of subjects, 28:0 PS, 34:0 PI and 14:0 LPS could be biomarkers capable of predicting COVID‐19 severity before symptom onset (Figure 7C). During the early phases (days 1–3 and days 4–6), several species of PC, PE and SM, as well as LPE and PI, had higher ROCs with lower *p* values, compared with the results for CRP and D‐dimer (Figure S11). During the days 7–9, days 10–12 and days 13–15, the CRP and D‐dimer levels could be used to differentiate the maximum severity accurately (Figures S12 and S13). The ranking of the parameters with the top three lowest *p* values is shown in Figure 7D.

### 2.9 Lipid modulations in COVID‐19 subjects resemble those seen in lipopolysaccharide‐induced septic mouse models but also exhibit several unique characteristics

Lastly, to understand the results more mechanistically, we investigated the modulations of the monitored lipids in the non‐COVID‐19 infectious disease group and in related mouse models. In the subjects with non‐COVID‐19 infectious diseases, we performed an OPLS‐DA to differentiate cases with confirmed bacterial infection (*n* = 80) from others (*n* = 29) (Figure S14A,B). Considering these results together with the results for the COVID‐19 subjects, the lipid modulations seen during COVID‐19 might have unique characteristics, since they resemble bacterial infection from the aspects of the elevations in 16:0 LPG and 16:1 LPG and the decrease in PC, whereas they resemble non‐bacterial infection from the aspect of the decrease in SM.

**FIGURE 8:**
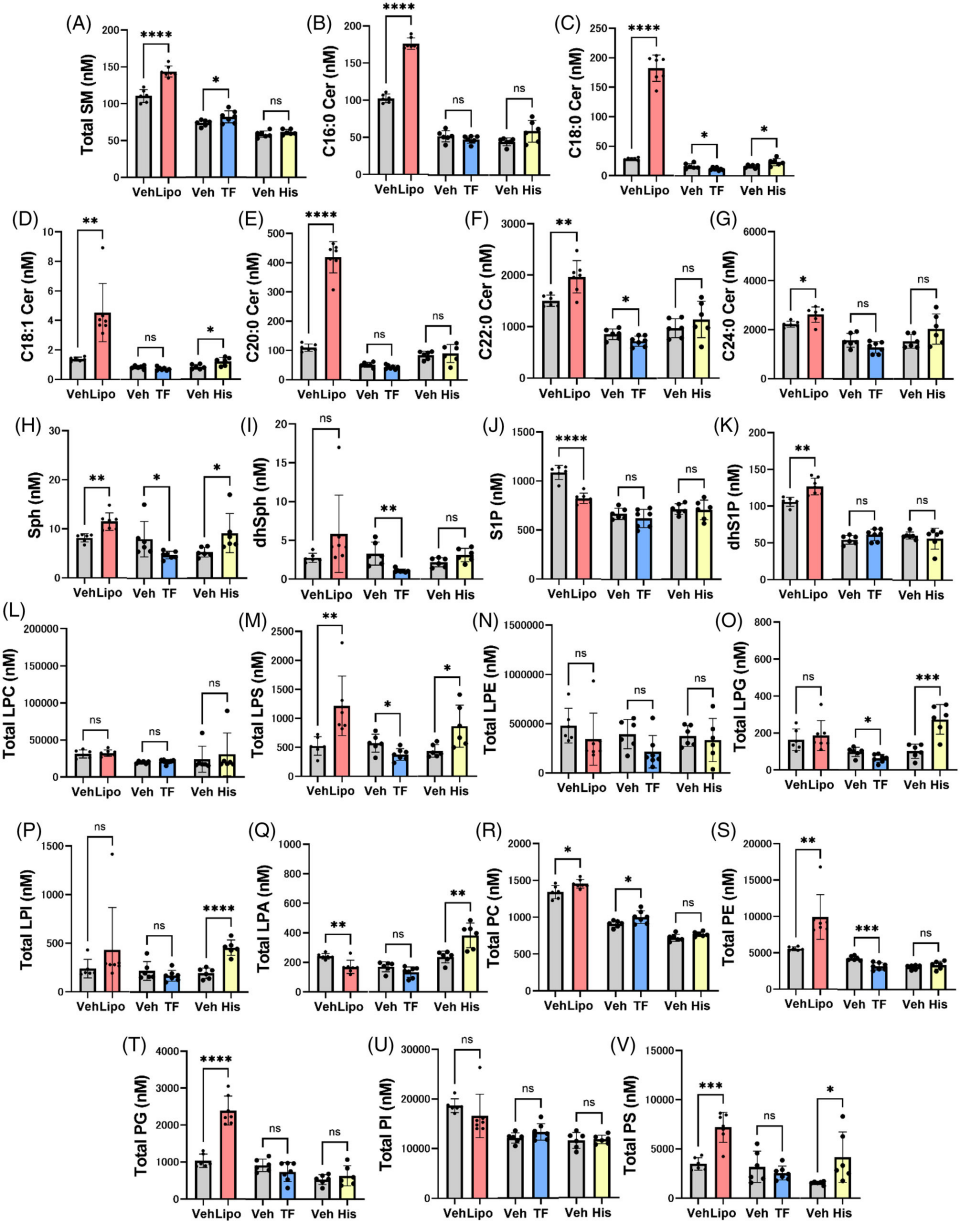
Modulations of sphingolipids and glycerophospholipids in mice administered lipopolysaccharides, tissue factor, or histone. Modulations of plasma sphingolipids and glycerophospholipids in mice treated with lipopolysaccharides (Lipo, *n* = 6–7), tissue factor (TF, *n* = 6–7), or histone (His, *n* = 6), prepared as described in the Methods section. The data are shown as the mean ± SD. *p < .05, **p < .01, ***p < .001, ****p < .0001

We next compared the modulations of lipids in three related mouse models using lipopolysaccharide (Lipo), tissue factor (TF) and histone (His). An overview of the lipid modulations is shown in Figure 8. Among the three models, the modulations of lipids in the lipopolysaccharide‐induced septic mouse models seemed most akin to those seen in severe COVID‐19. Among the modulations of lipids observed in severe COVID‐19, the elevations in SM, C16:0 ceramide, C18:0 ceramide, C18:1 ceramide, Sph, LPS, PC and PE and the reduction in S1P were observed in the mice treated with lipopolysaccharide, while elevations in SM and PC were observed in mice administered TF, and elevations in C18:0 ceramide, C18:1 ceramide, Sph, LPG and LPI were observed in mice injected with histone. To analyse the results in more detail, volcano plots were prepared and the results of an OPLS‐DA are shown (Figure S15). PC species with long unsaturated acyl chains and LPE decreased, while PC species with short acyl chains and most species of PE and SM increased in mice treated with lipopolysaccharide.

## 3 DISCUSSION

In this study, we performed targeted lipidomics analyses for sphingolipids and glycerophospholipids longitudinally with a rather large number of subjects and observed the dynamic changes in the serum samples of COVID‐19 subjects, depending on the time course and maximum severity as well as the molecular lipid species. We observed several modulations that were not concordant with previous reports. We think that these discrepancies can most likely be explained by differences in study design: compared with previous studies, our study examined the lipid levels in more detail and with longitudinal observations divided into short spans. We also examined the associations according to severity. We adopted this design because the time course and disease severity can have large effects on lipid modulation. Moreover, considering that targeted lipidomics is, in general, superior to untargeted lipidomics from the aspect of quantification, we believe that the present study provides information that will aid our understanding of lipid modulations in COVID‐19. The main results of the present study are summarised in Figure S16.

Severer COVID‐19 subjects had higher serum SM levels. Contrary to the present findings, several studies using untargeted lipidomics reported decreased SM levels depending on disease severity,^17^, ^18^, ^19^, ^20^ whereas one study using targeted lipidomics showed an increase in SM, with the exception of 32:2 SM.^21^ Ceramide modulations largely depend on the molecular species. The levels of C16:0 ceramide and C18:0 ceramide increased, whereas the levels of C18:1, C20:0, C22:0 and C24:0 ceramide decreased in the COVID‐19 subjects. Among the ceramide species that were monitored in the present study, Khodadoust et al., Gray et al. and Janneh et al. reported marked increases in C22:0, C18:0 and C24:0 ceramide, respectively,^22^, ^23^, ^24^ while Torretta et al. reported increases in C16:0, C18:0 and C20:0 ceramide and a decrease in C24:0 ceramide^18^ and Li et al. reported decreases in C22:0 and C24:0 ceramide.^25^ From the aspect of the association with disease severity, the present study showed increases in all the monitored ceramide species except C24:0 ceramide in maximum severity group 4, which seemed concordant with the previous reports^18^, ^22^, ^26^, ^27^, ^28^ except two reports demonstrating decreased ceramide levels in severer patients.^17^, ^25^ Regarding Sph and dhSph, we observed the increase in Sph in severer COVID‐19 patients, while the decrease in dhSph in milder COVID‐19 patients and the increase in Sph in severe COVID‐19 patients. The elevation of Sph and dhSph, especially in severe patients, was reported in a previous study using untargeted lipidomics^18^ and the decrease of Sph and dhSph in COVID‐19 patients was reported in one report with targeted lipidomics.^24^ Although serum S1P and dhS1P levels can hardly reflect their concentration in vivo, their levels were lower in COVID‐19 depending on the severity, which was concordant with the previous reports.^29^ Of course, the decreased platelet counts can largely explain these results, and the S1P and dhS1P levels were rather closely correlated with the platelet count (Figure 7A). However, the serum S1P levels were lower even in subjects with milder disease, asymptomatic subjects, or subjects before the onset of COVID‐19, which was concordant with the findings of previous studies using serum samples,^19^, ^24^ suggesting that some mechanisms are involved in the reduction in S1P levels caused by infection with SARS‐CoV‐2.

Regarding glycerophospholipids, for the LPC/PC axis, we observed a decrease in LPC in COVID‐19 subjects with mild disease, while an increase in PC was seen in COVID‐19 subjects with severe disease. Previous studies reported lower LPC levels,^17^, ^23^, ^26^ while several reports have shown higher LPC levels.^20^, ^21^ Some reports showed that subjects with severe disease reportedly had lower LPC levels,^17^, ^26^ whereas one paper reported the opposite.^21^ Contrary to the present results, most groups reported a decrease in PC,^17^, ^20^, ^21^, ^23^, ^25^, ^26^, ^27^, ^30^ with the exception of one group investigating asymptomatic subjects.^19^ These results might be explained by biphasic modulation depending on the molecular species of PC. Actually, we observed a severity‐dependent decrease in PC species with long and polyunsaturated acyl chains, as shown in Figure 3F,G. PC and LPC reflected and predicted the maximum disease severity, which is concordant with the results of previous studies.^26^, ^27^ Regarding the LPS/PS axis, although the PS levels could not be properly evaluated using serum samples (Figure S1), some LPS levels, such as 16:0 LPS and 18:0 LPS, decreased during the early phase, while 22:6 LPS increased during the late phase (Figure S8B,C and Figure 4E). The association with the maximum severity also depended on the LPS species. The 16:0 LPS and 18:0 LPS levels were lower in maximum severity group 4, whereas those of 18:2 LPS and 22:6 LPS were higher (Figures S8B,C and S9F; Figure 4E). Although the timing of the measurements was not clear, two studies reported that LPS levels were higher in COVID‐19,^21^, ^31^ while Hao et al. reported a decrease in LPS in asymptomatic subjects.^19^ In the present study, we observed lower LPE levels in COVID‐19 subjects with mild disease, while higher LPE levels were seen in COVID‐19 subjects with severe disease. The PE levels increased depending on the severity of the disease. The LPE levels have been reported to be higher^30^, ^32^ or lower^17^, ^20^ in COVID‐19, depending on the study design. The PE levels have also been reported to be higher in COVID‐19 subjects in some studies,^19^, ^21^, ^26^ while the PE levels were lower in others.^20^, ^23^, ^25^ Although previous studies did not report differences in molecular species, our studies revealed that LPE and PE behaved differently depending on the lipid species. For example, the association of PE with clinical parameters depended on its species (Figure 6), with 40:5 PE, 36:7 PE and 40:10 PE (Figure 3C and Figure S9H,I) increasing during the early phase and 18:3 LPE and 18:2 LPE (Figure 4D and Figure S8F) increasing during the late phase in maximum severity group 4. These species‐dependencies might at least partly explain the difference in findings between the present study and previous ones. For the LPG/PG axis and the LPI/PI axis, LPG decreased during the early phase and increased during the late phase and PG decreased, while LPI increased and PI decreased in COVID‐19 subjects. Previous reports have shown that PG decreases,^23^ PI decreases (especially in critically ill patients),^17^, ^20^, ^21^, ^25^ and LPI increases in COVID‐19 subjects with severe disease;^21^, ^26^ these findings were concordant with those of the present study. Some specific modulations were observed for several species: 38:4 PI, 40:5PI, 40:6PI increased and 40:10 PI decreased in this study (Figure 4F,H; Figure S10G,H). Among these axes, PI was negatively associated with the maximum severity during the early phase (Figure 5); this finding has not been previously reported.

Lipid modulations can reflect both conditions unique to infection with SARS‐CoV‐2 and those associated with the host immune response. The former modulations should differ from modulations observed in non‐COVID‐19 infectious diseases, while the latter should reflect pathological conditions related to the immune response. To understand the mechanism observed in the present study, we compared lipid modulations in COVID‐19 subjects with those in subjects with non‐COVID‐19 infectious diseases. Overall, we observed that most lipids were modulated in the same direction in both COVID‐19 subjects and subjects with non‐COVID‐19 infectious diseases; however, several lipids were modulated differently. Among sphingolipids, the elevation of C16:0 ceramide in severe COVID‐19 and the decreases in C18:1 ceramide and dhSph in mild COVID‐19 were specific to COVID‐19 (Figure 1). However, positive correlations of C18:1 ceramide with CRP and of C16:0 ceramide with D‐dimer were observed in both groups, suggesting that these differences might arise from some fundamental factors related to infection by a pathogen, rather than disease severity. Among glycerophospholipids, LPG and PG increased in subjects with non‐COVID‐19 infectious diseases, whereas LPG decreased during the early phase and increased during the late phase; PG decreased. LPI and PI were not modulated in subjects with non‐COVID‐19 infectious diseases, while LPI increased and PI decreased in COVID‐19 subjects (Figure 2). Actually, in our analysis of the correlations with clinical parameters, we observed several differences between COVID‐19 subjects and subjects with non‐COVID‐19 infectious diseases (Figure 7A,B), suggesting the presence of unique mechanisms of lipid dynamism in COVID‐19.

The administration of lipopolysaccharides in mice might model the cytokine storm that results from sepsis or suppressed‐fibrinolytic‐type DIC, whereas mice injected with TF are a model for enhanced fibrinolytic/balanced fibrinolytic DIC^33^ and mice treated with histone might model the pathological conditions of NETosis.^34^ Among these three mouse models (Figure 8), the modulations of SM, ceramides (excluding C24:0 ceramide), Sph, dhSph, LPC, PC, PE and PI were most akin to those occurring in mice treated with lipopolysaccharides; the modulations of LPS during the early phase were similar to those occurring in mice injected with TF; and the modulations of Sph, dhSph and LPI were similar to those occurring in mice administered histone. The downregulation of C24:0 ceramide and the modulations of LPG and PG were difficult to replicate in a mouse model. Collectively, the results suggest that most of the modulations observed in COVID‐19 might reflect conditions similar to septic shock caused by a cytokine storm but that the increases in LPS and LPI might be associated with enhanced fibrinolytic/balanced fibrinolytic DIC and NETosis, respectively.

Regarding the significance of sphingolipid modulation in pathogenesis, SM is associated with lipid rafts, which can promote virus entry across cellular surfaces.^35^, ^36^ SM is also important for the viral replication and release of several viruses.^37^, ^38^ Ceramide‐enriched domains also facilitate the transmembrane trafficking of viruses, such as adenovirus.^39^ Therefore, the increase in SM and the higher ceramide levels seen in subjects with severe COVID‐19 might be of significance in terms of the promotion of viral amplification. The decrease in C24:0 ceramide is more difficult to interpret. The physical characteristics of ceramides should depend on the length of their acyl chains,^40^ suggesting that ceramides with very long chains might alter cell membranes, thereby modulating the ease of viral entry. Actually, several previous studies measuring ceramide levels in COVID‐19 subjects have shown a reduction in ceramides with very long chains.^18^, ^25^ When discussing sphingolipid modulation from the aspect of the host immune response, C24:0 lactosylceramide, which is derived from C24:0 ceramide, possesses important properties in the natural immune response elicited by neutrophils.^41^, ^42^ Therefore, an enhanced immune response by neutrophils might expend C24:0 ceramide. According to the ceramide‐S1P rheostat theory, ceramides and sphingosine generally provoke apoptosis in the host, whereas S1P exerts pro‐survival effects. The association of sphingolipids with the maximum severity of the disease is almost concordant with the significance presumed by this theory.

Regarding the significance of glycerophospholipid modulation from the aspects of virus amplification, a recent report revealed that diacylphospholipids, such as PI, PC and PE, are enriched in the envelope of SARS‐CoV‐2,^43^ suggesting the possibility that increased PC and PE levels might accelerate the amplification of this virus and also the possible expenditure of PI during infection with SARS‐CoV‐2. From the aspect of host immune responses, the increased PC and LPC levels might increase the LPA levels through autotaxin. Although the roles of LPA in immune response and inflammation depend on the LPA receptors, considering that the levels of PC with less saturated acyl chains increased while the levels of PC with polysaturated acyl chains decreased, especially in subjects with severe COVID‐19 and the fact that polyunsaturated LPA is biased toward anti‐inflammatory LPA_3_,^44^, ^45^ these modulations might result in the overreaction of the immune response. Since several LPS species had strong negative correlations with CRP, especially during the early phase, LPS might mainly exert anti‐inflammatory properties during early infection, and the increase in 22:6 LPS during the late phase, especially in severe COVID‐19, might be a compensatory reaction. The increases in LPI and LPG, especially in severe COVID‐19, might facilitate inflammation, considering their biological activities through GPR55.^14^ Although the roles of LPE and PE in infection remain unknown, considering that LPE might possess anti‐inflammatory properties on macrophages,^15^ the elevation of the LPE/PE axis in severe COVID‐19 might also be a compensatory reaction.

Since several characteristics of patients are risk factors for severe COVID‐19, the characteristics, such as sex, age and the presence of hypertension, were different, especially among the control or severity 1 group and severer groups (Tables S1 and S2). To remove potential confounding factors, we analysed the results longitudinally with the paired Wilcoxon signed‐rank test in each severity group, using the results at day 25–40 as control, although the modulations of the lipids might be prolonged to some degree as reported previously.^24^, ^46^ As shown in Figures S17–S20, most of the main results were confirmed; ceramides decreased in milder groups while increased in the severity 4 group; SM, Sph and dhSph increased in COVID‐19; LPC decreased in COVID‐19; and LPG and LPI decreased in milder patients while increased in the severity 4 group. Regarding S1P and dhS1P, we confirmed that they decreased at least in severity 4 group. Regarding PC, LPE and PG, considering that their association with sex, age and the presence of hypertension in healthy subjects could not explain their modulations in severer patients, the modulations shown in Figure 2 might not be derived from confounding factors. Although the elevation of PE in the severity 4 group might be derived from potential confounding factors, since the presence of hypertension and age were positively correlated with serum PE levels in healthy subjects. However, considering that the elevation of PE was observed in other severity groups and the characteristics of subjects were not so different among severity 2, 3 and 4 group, we think the elevation of PE was prolonged to day 25–40 in severity 4 group, which might blur the elevation of PE in the analyses shown in Figure S19. Another limitation is that since this is an observational study, we could not elucidate the underlining mechanisms for the modulations of the lipids. Reportedly, Group IIA secreted phospholipase A_2_ is associated with COVID‐19 mortality.^31^ The present results of increased levels of LPG and LPI and decreased levels of PG and PI were concordant with the elevation of phospholipase A_2_, which was proposed in the previous paper.^31^ Although the modulations of lipids could not be simply explained, phospholipases outside and inside the cells might be involved in the modulations of glycerophospholipids. Actually, we recently reported the elevation of phosphatidylserine‐specific phospholipase A_1_ in COVID‐19.^47^

In summary, we observed the dynamic modulations of sphingolipids and glycerophospholipids in serum samples from patients with COVID‐19, some of which were dependent on the time course and severity. We believe that an understanding of the dynamic modulations of these lipids in COVID‐19 will help us to understand the involvement of lipids in the infectious process of SARS‐CoV‐2 and the host response. These results may also prompt researchers to further investigate the associations of sphingolipids and glycerophospholipids with COVID‐19 to develop laboratory testing for the prediction of maximum severity and/or novel agents to suppress the aggravation of COVID‐19.

## 4 METHODS

### 4.1 Samples

We collected residual serum samples available after routine clinical testing from 215 subjects who had been diagnosed as having COVID‐19 based on the results of RT‐PCR assays performed between April 2020 and June 2021. None of the subjects enrolled in the present study had been vaccinated against SARS‐CoV‐2 at the time of blood sampling. One sample was collected from one subject at as many of the following eight intervals as possible: 1–5 days before symptom onset (Pre), and days 1–3, days 4–6, days 7–9, days 10–12, days 13–15, days 16–18, days 19–24 and days 25–40 after symptom onset. The subjects were classified into four groups according to the maximum disease severity: maximum severity group 1 (did not require oxygen supplementation), maximum severity group 2 (required oxygen supplementation at low flow rates of under 4 L/min via a nasal cannula), maximum severity group 3 (required oxygen supplementation at relatively high flow rates, but did not require mechanical ventilatory support), and maximum severity group 4 (required mechanical ventilatory support). The characteristics of all the subjects and the subjects analysed at specific time points are described in Tables S1 and S2, respectively. The time courses for CRP and D‐dimer are shown in Figure S21. As a control, we collected 115 serum samples from volunteers without infectious diseases and 109 serum samples from subjects with infectious diseases other than COVID‐19 and in whom COVID‐19 had been ruled out by the results of an RT‐PCR test. The day after the onset of COVID‐19 was determined based on the date of symptom onset reported by each subject.

The current study was performed in accordance with the ethical guidelines laid down in the Declaration of Helsinki. Written informed consent for sample analysis was obtained from some of the subjects. For the remaining participants from whom written informed consent could not be obtained (because of hospital discharge or transfer to another hospital), informed consent was obtained in the form of an opt‐out on a website, as follows. Subjects were informed of the study on the website, and those who were unwilling to be enrolled in our study were excluded. The study design was approved by The University of Tokyo Medical Research Center Ethics Committee (2602 and 2020206NI).

### 4.2 Animal experiments

To investigate the effects of lipopolysaccharides on lipids, ten‐week‐old C57BL/6 mice, purchased from CLEA Japan (Tokyo, Japan), were injected with lipopolysaccharides (124‐05151; WAKO Pure Chemical Industries, Osaka, Japan) at a dose of 10 mg/kg BW intraperitoneally. Plasma samples were collected after 24 h. To investigate the effects of TF, ten‐week‐old mice were intravenously administered with 5 μL/g BW of TF‐containing solution, as prepared by diluting Recombiplastin (0020002950; Instrumentation Laboratory) in phosphate‐buffered saline at a ratio of 1:80, as previously reported.^48^ Plasma samples were collected after 24 h. To investigate the effects of histone, ten‐week‐old mice were intravenously administered with histone from calf thymus (H9250; Sigma‐Aldrich). Plasma samples were collected after 4 h. All the animal experiments were conducted in accordance with the guidelines for Animal Care and were approved by the animal committee of The University of Tokyo (protocols P13‐036 and P17‐075).

### 4.3 LC‐MS/MS measurements of glycerolysophospholipids, diacylphospholipids and sphingolipids

We measured the levels of the lipid mediators using four independent LC‐MS/MS methods and an LC8060 system consisting of a quantum ultra‐triple quadrupole mass spectrometer (Shimadzu, Japan) as described and validated previously.^49^, ^50^, ^51^, ^52^

### 4.4 Statistical analysis

The results were expressed in dot plots. The data were analysed using SPSS (Chicago, IL) or MetaboAnalyst 5.0 (https://www.metaboanalyst.ca/). To examine differences in the lipid time courses among the healthy subjects and the COVID‐19 maximum severity groups 1, 2, 3 and 4, we evaluated the significant difference using the Kruskal–Wallis test, followed by the Steel–Dwass test as a post‐hoc test. To examine differences between healthy subjects and subjects with non‐COVID‐19 infectious diseases, we used the Mann–Whitney U test. To examine differences in the lipid levels longitudinally between specific time points and day 25–40 in a specific maximum severity group, we used the paired Wilcoxon signed‐rank test (Figures S17–S20). An OPLS‐DA was performed using MetaboAnalyst to explore variables capable of differentiating COVID‐19 subjects from healthy subjects (Figure 3A) and the non‐COVID‐19 group (Figure 4A). The time courses for the VIP scores and the T scores are shown in the heat maps. For the correlation studies, the Kendall rank correlation was used to examine the association of the lipids or clinical data (WBC, neutrophil count [neutro], lymphocyte count [lymph], monocyte count [mono], eosinophil count [eosino], basophil count [baso], RBC, platelet count, MCV, MPV, RDW, PDW, CRP, D‐dimer, albumin [Alb], AST, ALT, Cre, PT‐INR, APTT and fibrinogen [Fbg]) with the maximum severity of the disease (Figure 5), considering age, sex and the presence of diabetes, hypertension and current smoking as covariates of interest. The Spearman rank correlation was used to examine the associations between lipids and clinical data. A receiver operating characteristic (ROC) curve analysis was used to investigate the lipids, CRP or D‐dimer, which predicts the maximum severity of the disease (Figure 7). To compare the lipids between plasma samples taken under strict conditions as described previously^16^ and the routine serum samples, we used a paired *t*‐test (Figure S1); to evaluate lipid differences in the mice experiments, we used the student's *t*‐test (Figure 8). The graphic figures were prepared using Graphpad Prism 9 (GraphPad Software, San Diego, CA) or MetaboAnalyst. *P* values of less than .05 were deemed as denoting statistical significance in all the analyses.

## CONFLICT OF INTERESTS

The authors declare that there is no conflict of interest that could be perceived as prejudicing the impartiality of the research reported.

## Supporting information

Supporting information

## References

[1] Sander WJ , O'Neill HG , Pohl CH . Prostaglandin E2 as a modulator of viral infections. Front Physiol. 2017;8:89. 10.3389/fphys.2017.00089 28261111 PMC5306375

[2] Kalinski P . Regulation of immune responses by prostaglandin E2. J Immunol. 2012;188(1):21‐8. 10.4049/jimmunol.1101029 22187483 PMC3249979

[3] Maceyka M , Payne SG , Milstien S , Spiegel S . Sphingosine kinase, sphingosine‐1‐phosphate, and apoptosis. Biochim Biophys Acta. 2002;1585(2‐3):193‐201. 10.1016/s1388-1981(02)00341-4 12531554

[4] Burg N , Salmon JE , Hla T . Sphingosine 1‐phosphate receptor‐targeted therapeutics in rheumatic diseases. Nat Rev Rheumatol. 2022;18(6):335‐351. 10.1038/s41584-022-00784-6 35508810 PMC9641499

[5] Obeid LM , Linardic CM , Karolak LA , Hannun YA . Programmed cell death induced by ceramide. Science. 1993;259(5102):1769‐71. 10.1126/science.8456305 8456305

[6] Vandanmagsar B , Youm YH , Ravussin A , et al. The NLRP3 inflammasome instigates obesity‐induced inflammation and insulin resistance. Nat Med. 2011;17(2):179‐88. 10.1038/nm.2279 21217695 PMC3076025

[7] Bartke N , Hannun YA . Bioactive sphingolipids: metabolism and function. J Lipid Res. 2009;50 suppl:S91‐6. 10.1194/jlr.R800080-JLR200 19017611 PMC2674734

[8] Makide K , Uwamizu A , Shinjo Y , et al. Novel lysophosphoplipid receptors: their structure and function. J Lipid Res. 2014;55(10):1986‐95. 10.1194/jlr.R046920 24891334 PMC4173991

[9] Gustin C , Van Steenbrugge M , Raes M . LPA modulates monocyte migration directly and via LPA‐stimulated endothelial cells. Am J Physiol Cell Physiol. 2008;295(4):C905‐14. 10.1152/ajpcell.00544.2007 18632732

[10] Pei S , Xu C , Pei J , et al. Lysophosphatidic acid receptor 3 suppress neutrophil extracellular traps production and thrombosis during sepsis. Front Immunol. 2022;13:844781. 10.3389/fimmu.2022.844781 35464399 PMC9021375

[11] Hu J , Oda SK , Shotts K , et al. Lysophosphatidic acid receptor 5 inhibits B cell antigen receptor signaling and antibody response. J Immunol. 2014;193(1):85‐95. 10.4049/jimmunol.1300429 24890721 PMC4128188

[12] Barnes MJ , Li CM , Xu Y , An J , Huang Y , Cyster JG . The lysophosphatidylserine receptor GPR174 constrains regulatory T cell development and function. J Exp Med. 2015;212(7):1011‐20. 10.1084/jem.20141827 26077720 PMC4493414

[13] Gurusamy M , Tischner D , Shao J , et al. G‐protein‐coupled receptor P2Y10 facilitates chemokine‐induced CD4 T cell migration through autocrine/paracrine mediators. Nat Commun. 2021;12(1):6798. 10.1038/s41467-021-26882-9 34815397 PMC8611058

[14] Kurano M , Kobayashi T , Sakai E , Tsukamoto K , Yatomi Y . Lysophosphatidylinositol, especially albumin‐bound form, induces inflammatory cytokines in macrophages. FASEB J. 2021;35(6):e21673. 10.1096/fj.202100245R 34042213

[15] Park SJ , Im DS . 2‐Arachidonyl‐lysophosphatidylethanolamine induces anti‐inflammatory effects on macrophages and in carrageenan‐induced paw edema. Int J Mol Sci. 2021;22(9):4865. 10.3390/ijms22094865 34064436 PMC8125189

[16] Nakamura K , Kishimoto T , Ohkawa R , et al. Suppression of lysophosphatidic acid and lysophosphatidylcholine formation in the plasma in vitro: proposal of a plasma sample preparation method for laboratory testing of these lipids. Anal Biochem. 2007;367(1):20‐7. 10.1016/j.ab.2007.05.004 17568554

[17] Dei Cas M , Ottolenghi S , Morano C , et al. Link between serum lipid signature and prognostic factors in COVID‐19 patients. Sci Rep. 2021;11(1):21633. 10.1038/s41598-021-00755-z 34737330 PMC8568966

[18] Torretta E , Garziano M , Poliseno M , et al. Severity of COVID‐19 patients predicted by serum sphingolipids signature. Int J Mol Sci. 2021;22(19):10198. 10.3390/ijms221910198 34638539 PMC8508132

[19] Hao Y , Zhang Z , Feng G , et al. Distinct lipid metabolic dysregulation in asymptomatic COVID‐19. iScience. 2021;24(9):102974. 10.1016/j.isci.2021.102974 34396083 PMC8356725

[20] Barberis E , Timo S , Amede E , et al. Large‐scale plasma analysis revealed new mechanisms and molecules associated with the host response to SARS‐CoV‐2. Int J Mol Sci. 2020;21(22):8623. 10.3390/ijms21228623 33207699 PMC7696386

[21] Song JW , Lam SM , Fan X , et al. Omics‐driven systems interrogation of metabolic dysregulation in COVID‐19 pathogenesis. Cell Metab. 2020;32(2):188‐202 e5. 10.1016/j.cmet.2020.06.016 PMC7311890 32610096

[22] Khodadoust MM . Inferring a causal relationship between ceramide levels and COVID‐19 respiratory distress. Sci Rep. 2021;11(1):20866. 10.1038/s41598-021-00286-7 34675292 PMC8531370

[23] Gray N , Lawler NG , Zeng AX , et al. Diagnostic potential of the plasma lipidome in infectious disease: application to acute SARS‐CoV‐2 infection. Metabolites. 2021;11(7):467. 10.3390/metabo11070467 34357361 PMC8306636

[24] Janneh AH , Kassir MF , Dwyer CJ , et al. Alterations of lipid metabolism provide serologic biomarkers for the detection of asymptomatic versus symptomatic COVID‐19 patients. Sci Rep. 2021;11(1):14232. 10.1038/s41598-021-93857-7 34244584 PMC8270895

[25] Li Y , Hou G , Zhou H , et al. Multi‐platform omics analysis reveals molecular signature for COVID‐19 pathogenesis, prognosis and drug target discovery. Signal Transduct Target Ther. 2021;6(1):155. 10.1038/s41392-021-00508-4 33859163 PMC8047575

[26] Wu P , Chen D , Ding W , et al. The trans‐omics landscape of COVID‐19. Nat Commun. 2021;12(1):4543. 10.1038/s41467-021-24482-1 34315889 PMC8316550

[27] Sindelar M , Stancliffe E , Schwaiger‐Haber M , et al. Longitudinal metabolomics of human plasma reveals prognostic markers of COVID‐19 disease severity. Cell Rep Med. 2021;2(8):100369. 10.1016/j.xcrm.2021.100369 34308390 PMC8292035

[28] Caterino M , Gelzo M , Sol S , et al. Dysregulation of lipid metabolism and pathological inflammation in patients with COVID‐19. Sci Rep. 2021;11(1):2941. 10.1038/s41598-021-82426-7 33536486 PMC7859398

[29] Marfia G , Navone S , Guarnaccia L , et al. Decreased serum level of sphingosine‐1‐phosphate: a novel predictor of clinical severity in COVID‐19. EMBO Mol Med. 2021;13(1):e13424. doi:10.15252/emmm.202013424 33190411 PMC7744841

[30] Wu D , Shu T , Yang X , et al. Plasma metabolomic and lipidomic alterations associated with COVID‐19. Natl Sci Rev. 2020;7(7):1157‐1168. 10.1093/nsr/nwaa086 34676128 PMC7197563

[31] Snider JM , You JK , Wang X , et al. Group IIA secreted phospholipase A2 is associated with the pathobiology leading to COVID‐19 mortality. J Clin Invest. 2021;131(19):e149236. 10.1172/JCI149236 34428181 PMC8483752

[32] Castane H , Iftimie S , Baiges‐Gaya G , et al. Machine learning and semi‐targeted lipidomics identify distinct serum lipid signatures in hospitalized COVID‐19‐positive and COVID‐19‐negative patients. Metabolism. 2022;131:155197. 10.1016/j.metabol.2022.155197 35381232 PMC8976580

[33] Asakura H . Classifying types of disseminated intravascular coagulation: clinical and animal models. J Intensive Care. 2014;2(1):20. 10.1186/2052-0492-2-20 25520834 PMC4267600

[34] Xu J , Zhang X , Pelayo R , et al. Extracellular histones are major mediators of death in sepsis. Nat Med. 2009;15(11):1318‐21. 10.1038/nm.2053 19855397 PMC2783754

[35] Otsuki N , Sakata M , Saito K , et al. Both sphingomyelin and cholesterol in the host cell membrane are essential for rubella virus entry. J Virol. 2018;92(1):e01130‐17. 10.1128/JVI.01130-17 29070689 PMC5730775

[36] Audi A , Soudani N , Dbaibo G , Zaraket H . Depletion of host and viral sphingomyelin impairs influenza virus infection. Front Microbiol. 2020;11:612. 10.3389/fmicb.2020.00612 32425895 PMC7203554

[37] Sakamoto H , Okamoto K , Aoki M , et al. Host sphingolipid biosynthesis as a target for hepatitis C virus therapy. Nat Chem Biol. 2005;1(6):333‐7. 10.1038/nchembio742 16408072

[38] Tafesse FG , Sanyal S , Ashour J , et al. Intact sphingomyelin biosynthetic pathway is essential for intracellular transport of influenza virus glycoproteins. Proc Natl Acad Sci U S A. 2013;110(16):6406‐11. 10.1073/pnas.1219909110 23576732 PMC3631694

[39] Zha X , Pierini LM , Leopold PL , Skiba PJ , Tabas I , Maxfield FR . Sphingomyelinase treatment induces ATP‐independent endocytosis. J Cell Biol. 1998;140(1):39‐47. 10.1083/jcb.140.1.39 9425152 PMC2132600

[40] Silva L , de Almeida RF , Fedorov A , Matos AP , Prieto M . Ceramide‐platform formation and ‐induced biophysical changes in a fluid phospholipid membrane. Mol Membr Biol. 2006;23(2):137‐48. 10.1080/09687860500439474 16754357

[41] Iwabuchi K , Prinetti A , Sonnino S , et al. Involvement of very long fatty acid‐containing lactosylceramide in lactosylceramide‐mediated superoxide generation and migration in neutrophils. Glycoconj J. 2008;25(4):357‐74. 10.1007/s10719-007-9084-6 18041581

[42] Nakayama H , Yoshizaki F , Prinetti A , et al. Lyn‐coupled LacCer‐enriched lipid rafts are required for CD11b/CD18‐mediated neutrophil phagocytosis of nonopsonized microorganisms. J Leukoc Biol. 2008;83(3):728‐41. 10.1189/jlb.0707478 18055569

[43] Saud Z , Tyrrell VJ , Zaragkoulias A , et al. The SARS‐CoV2 envelope differs from host cells, exposes pro‐coagulant lipids, and is disrupted in vivo by oral rinses. J Lipid Res. 2022;63(6):100208. 10.1016/j.jlr.2022.100208 35436499 PMC9010312

[44] Fujiwara Y , Sardar V , Tokumura A , et al. Identification of residues responsible for ligand recognition and regioisomeric selectivity of lysophosphatidic acid receptors expressed in mammalian cells. J Biol Chem. 2005;280(41):35038‐50. 10.1074/jbc.M504351200 16115890

[45] Hama K , Aoki J . LPA(3), a unique G protein‐coupled receptor for lysophosphatidic acid. Prog Lipid Res. 2010;49(4):335‐42. 10.1016/j.plipres.2010.03.001 20230855

[46] Bizkarguenaga M , Bruzzone C , Gil‐Redondo R , et al. Uneven metabolic and lipidomic profiles in recovered COVID‐19 patients as investigated by plasma NMR metabolomics. NMR Biomed. 2022;35(2):e4637. 10.1002/nbm.4637 34708437 PMC8646702

[47] Shimura T , Kurano M , Okamoto K , et al. Increase in serum levels of phosphatidylserine‐specific phospholipase A1 in COVID‐19 patients. Cell Mol Immunol. 2021;18(9):2275‐2277. 10.1038/s41423-021-00744-2 34321622 PMC8316701

[48] Castillo MM , Yang Q , Zhan M , et al. Maintaining extraembryonic expression allows generation of mice with severe tissue factor pathway inhibitor deficiency. Blood Adv. 2019;3(3):489‐498. 10.1182/bloodadvances.2018018853 30755437 PMC6373739

[49] Morita Y , Kurano M , Sakai E , et al. Analysis of urinary sphingolipids using liquid chromatography‐tandem mass spectrometry in diabetic nephropathy. J Diabetes Investig. 2020;11(2):441‐449. 10.1111/jdi.13154 PMC7078086 31580528

[50] Sakai E , Kurano M , Morita Y , Aoki J , Yatomi Y . Establishment of a measurement system for sphingolipids in the cerebrospinal fluid based on liquid chromatography‐tandem mass spectrometry, and its application in the diagnosis of carcinomatous meningitis. J Appl Lab Med. 2020;5(4):656‐670. 10.1093/jalm/jfaa022 32407524

[51] Morita Y , Kurano M , Sakai E , et al. Evaluation of lysophospholipid measurement in cerebrospinal fluid samples using liquid chromatography‐tandem mass spectrometry. Lipids. 2019;54(8):487‐500. 10.1002/lipd.12175 31243768

[52] Kurano M , Yasukawa K , Ikeda H , Aoki J , Yatomi Y . Redox state of albumin affects its lipid mediator binding characteristics. Free Radic Res. 2019;53(8):892‐900. 10.1080/10715762.2019.1641603 31357898

