# Supplementary material for: Dynamic modulations of sphingolipids and glycerophospholipids in COVID‐19
Source: Clin Transl Med. 2022 Oct 10;12(10):e1069. doi: 10.1002/ctm2.1069 (PMC9549873; doi:10.1002/ctm2.1069)
Supplement: Supplementary file 1 — Supporting information [file CTM2-12-e1069-s001.docx]

**Supplemental Table S1. Characteristics of the subjects**

|  | Healthy subjects | Non-COVID infectious diseases | Maximum severity group 1 | Maximum severity group 2 | Maximum severity group 3 | Maximum severity group 4 | Asymptomatic |  |
| --- | --- | --- | --- | --- | --- | --- | --- | --- |
| Number of subjects | 115 | 109 | 68 | 61 | 38 | 36 | 12 |  |
| Age | 50.8 ± 16.5 | 62.1 ± 18.9*** | 51.8 ± 18.5 | 63.8 ± 14.0*** | 66.6 ± 13.5*** | 69.4 ± 12.3*** | 60.7 ± 22.9 | 1 vs. 2**  1 vs. 3**  1 vs. 4*** |
| Sex (male %) | 73.0 | 65.1 | 57.4 | 67.2 | 76.3 | 80.6 | 41.7 | *p* = .044 |
| Diabetes (%) | 38.3 | 37.6 | 22.1 | 27.9 | 47.4 | 36.1 | 16.7 | N.S. |
| Hypertension (%) | 27.0 | 42.2 | 30.9 | 50.8 | 57.9 | 50.0 | 41.7 | *p* = .002 |
| Current smoking (%) | 21.7 | 24.8 | 14.7 | 18.0 | 13.2 | 19.4 | 8.3 | N.S. |
| Use of steroids (%) |  |  | 10.3 | 63.9 | 86.8 | 88.9 | 0.0 | *p* < .001 |
| Use of remdesivir (%) |  |  | 5.9 | 53.3 | 68.4 | 33.3 | 0.0 | *p* < .001 |

Age: **p* < .05, ***p* < .01, ****p* < .001 vs. healthy subjects. Other parameters were examined using the χ^2^ test. The *p* values are shown.

**Supplemental Table S2. Subjects analyzed at specific time points**

**A. Before symptom onset**

| Maximum severity | 1 | 2 | 3 | 4 |  |
| --- | --- | --- | --- | --- | --- |
| Number of subjects | 13 | 4 |  | 1 |  |
| Age | 58.8 ± 19.7 | 53.0 ± 18.0 |  | 76.0 | N.S. |
| Sex (male %) | 53.8 | 50.0 |  | 1.0 | N.S. |
| Diabetes (%) | 38.5 | 25.0 |  | 0.0 | N.S. |
| Hypertension (%) | 46.2 | 75.0 |  | 0.0 | N.S. |
| Current smoking (%) | 7.7 | 0.0 |  | 0.0 | N.S. |
| Use of steroids (%) | 7.7 | 75.0 |  | 0.0 | N.S. |
| Use of remdesivir (%) | 0.0 | 25.0 |  | 0.0 | N.S. |

**B. Days 1–3**

| Maximum severity | 1 | 2 | 3 | 4 |  |
| --- | --- | --- | --- | --- | --- |
| Number of subjects | 22 | 5 | 1 | 3 |  |
| Age | 50 ± 19.8 | 43.8±17.7 | 78.0 | 81.7±1.89 | N.S. |
| Sex (male %) | 50.0 | 60.0 | 100.0 | 66.7 |  |
| Diabetes (%) | 31.8 | 20.0 | 0.0 | 33.3 |  |
| Hypertension (%) | 36.4 | 40.0 | 100.0 | 33.3 |  |
| Current smoking (%) | 13.6 | 0.0 | 0.0 | 0.0 |  |
| Use of steroids (%) | 0.0 | 60.0 | 100.0 | 100.0 | *p* < .001 |
| Use of remdesivir (%) | 0.0 | 60.0 | 100.0 | 0.0 | *p* < .001 |

**C. Days 4–6**

| Maximum severity | 1 | 2 | 3 | 4 |  |
| --- | --- | --- | --- | --- | --- |
| Number of subjects | 40 | 35 | 4 | 12 |  |
| Age | 48.2 ± 18.4 | 64.3 ± 15.5** | 71.5 ± 7.8 | 79.3 ± 8.2*** | 1 vs. 2***  1 vs. 4*** |
| Sex (male %) | 62.5 | 64.7 | 50.0 | 66.7 |  |
| Diabetes (%) | 30.0 | 32.4 | 50.0 | 50.0 |  |
| Hypertension (%) | 32.5 | 55.9 | 75.0 | 41.7 | *p* = .011 |
| Current smoking (%) | 12.5 | 20.6 | 25.0 | 16.7 |  |
| Use of steroids (%) | 10.0 | 67.6 | 100.0 | 75.0 | *p* < .001 |
| Use of remdesivir (%) | 7.5 | 41.2 | 75.0 | 41.7 | *p* = .001 |

**D. Days 7–9**

| Maximum severity | 1 | 2 | 3 | 4 |  |
| --- | --- | --- | --- | --- | --- |
| Number of subjects | 45 | 48 | 16 | 17 |  |
| Age | 51.4 ± 18.8 | 63.8 ± 14.8*** | 67.8 ± 14.7** | 74.1 ± 10.0*** | 1 vs. 2*  1 vs. 3*  1 vs. 4*** |
| Sex (male %) | 55.6 | 68.8 | 81.3 | 62.5 |  |
| Diabetes (%) | 31.1 | 29.2 | 43.8 | 47.1 |  |
| Hypertension (%) | 33.3 | 52.1 | 56.3 | 35.3 | *p* = .014 |
| Current smoking (%) | 11.1 | 22.9 | 12.5 | 23.5 |  |
| Use of steroids (%) | 15.6 | 66.7 | 93.8 | 82.4 | *p* < .001 |
| Use of remdesivir (%) | 11.1 | 52.1 | 81.3 | 47.1 | *p* < .001 |

**E. Days 10–12**

| Maximum severity | 1 | 2 | 3 | 4 |  |
| --- | --- | --- | --- | --- | --- |
| Number of subjects | 34 | 52 | 33 | 29 |  |
| Age | 52.8 ± 16.9 | 62.8 ± 12.3*** | 66.6 ± 14.2*** | 70.4 ± 10.3*** | 1 vs. 3***  1 vs. 4*** |
| Sex (male %) | 58.8 | 73.1 | 78.8 | 69.0 |  |
| Diabetes (%) | 20.0 | 28.8 | 45.5 | 44.8 |  |
| Hypertension (%) | 34.3 | 50.0 | 60.6 | 65.5 | *p* < .001 |
| Current smoking (%) | 14.3 | 26.9 | 15.2 | 24.1 |  |
| Use of steroids (%) | 17.1 | 63.5 | 87.9 | 96.6 | *p* < .001 |
| Use of remdesivir (%) | 8.6 | 46.2 | 69.7 | 41.4 | *p* < .001 |

**F. Days 13–15**

| Maximum severity | 1 | 2 | 3 | 4 |  |
| --- | --- | --- | --- | --- | --- |
| Number of subjects | 21 | 38 | 31 | 35 |  |
| Age | 57.9 ± 15.9 | 65.3 ± 11.8*** | 66.1 ± 14.3*** | 67.5 ± 11.8*** |  |
| Sex (male %) | 57.1 | 65.8 | 83.9 | 80.0 |  |
| Diabetes (%) | 33.3 | 18.4 | 45.2 | 42.9 |  |
| Hypertension (%) | 47.6 | 44.7 | 61.3 | 48.6 | *p* = .003 |
| Current smoking (%) | 23.8 | 21.1 | 16.1 | 22.9 |  |
| Use of steroids (%) | 14.3 | 57.9 | 90.3 | 91.4 | *p* < .001 |
| Use of remdesivir (%) | 9.5 | 44.7 | 67.7 | 40.0 | *p* = .001 |

**G. Days 16–18**

| Maximum severity | 1 | 2 | 3 | 4 |  |
| --- | --- | --- | --- | --- | --- |
| Number of subjects | 10 | 23 | 28 | 28 |  |
| Age | 60.5 ± 18.6 | 67.9 ± 13.9*** | 69.3 ± 11.8*** | 66.5 ± 11.0*** |  |
| Sex (male %) | 70.0 | 60.9 | 75.0 | 80.0 |  |
| Diabetes (%) | 10.0 | 21.7 | 42.9 | 78.6 |  |
| Hypertension (%) | 30.0 | 52.2 | 64.3 | 35.7 | *p* = .002 |
| Current smoking (%) | 20.0 | 13.0 | 14.3 | 46.4 |  |
| Use of steroids (%) | 0.0 | 47.8 | 85.7 | 28.6 | *p* < .001 |
| Use of remdesivir (%) | 0.0 | 30.4 | 64.3 | 92.9 | *p* = .001 |

**H. Days 19–24**

| Maximum severity | 1 | 2 | 3 | 4 |  |
| --- | --- | --- | --- | --- | --- |
| Number of subjects | 9 | 17 | 21 | 28 |  |
| Age | 65.3 ± 11.9 | 64.2 ± 15.3 | 70.3 ± 11.9*** | 67.2 ± 12.1*** |  |
| Sex (male %) | 100.0 | 64.7 | 81.0 | 85.7 |  |
| Diabetes (%) | 22.2 | 29.4 | 52.4 | 46.4 |  |
| Hypertension (%) | 55.6 | 52.9 | 66.7 | 53.6 | *p* = .001 |
| Current smoking (%) | 11.1 | 5.9 | 14.3 | 28.6 |  |
| Use of steroids (%) | 11.1 | 41.2 | 81.0 | 85.7 | *p* < .001 |
| Use of remdesivir (%) | 0.0 | 11.8 | 57.1 | 32.1 | *p* = .003 |

**I. Days 25–40**

| Maximum severity | 1 | 2 | 3 | 4 |  |
| --- | --- | --- | --- | --- | --- |
| Number of subjects | 31 | 49 | 25 | 21 |  |
| Age | 53.3 ± 17.9 | 63.1 ± 12.8*** | 65.4 ± 14.6** | 63.9 ± 11.0*** |  |
| Sex (male %) | 58.1 | 68.8 | 80.0 | 85.7 |  |
| Diabetes (%) | 25.8 | 24.5 | 48.0 | 52.4 |  |
| Hypertension (%) | 32.3 | 44.9 | 68.0 | 52.4 | *p* = .001 |
| Current smoking (%) | 12.9 | 24.5 | 12.0 | 33.3 |  |
| Use of steroids (%) | 9.7 | 59.2 | 80.0 | 90.5 | *p* < .001 |
| Use of remdesivir (%) | 3.2 | 44.9 | 60.0 | 28.6 | *p* < .001 |

Age: **p* < .05, ***p* < .01, ****p* < .001 vs. healthy subjects. Other parameters were examined using the χ^2^ test. The *p* values are shown.

**Supplemental Figure S1. Differences in lipid concentrations between plasma samples prepared under strict conditions and serum samples.**

**Supplemental Figure S1.** Comparison of glycerophospholipids (A–K) and sphingolipids (L–V) in plasma samples prepared under strict conditions and serum samples collected from healthy subjects at the same time points (n = 10). Paired *t*-tests were performed. **p* < .05, ***p* < .01, ****p* < .001, *****p* < .0001.

**Supplemental Figure S2. Modulations of sphingolipids and glycerophospholipids during the time course of asymptomatic COVID-19.**

**Supplemental Figure S2.** Serum total glycerophospholipid levels were measured longitudinally in asymptomatic COVID-19 subjects (n = 12). ‘Day’ shows the number of days after the diagnosis of infection with SARS-CoV-2 using an RT-PCR test. **p* < .05, ***p* < .01.

**Supplemental Figure S3. PCA to identify significant lipids capable of differentiating COVID-19 from healthy subjects.**

**Supplemental Figure S3.** A PCA using the measured lipid levels (excluding PS, S1P, and dhS1P) and the clinical phenotypes was performed to identify significant lipids capable of differentiating COVID-19 subjects from healthy subjects. The loading scatter plots are shown.

**Supplemental Figure S4. OPLS-DA** **to investigate significant lipid modulations characteristic of COVID-19 subjects before symptom onset, on days 1**–**3, and on days 4**–**6.**

**Supplemental Figure S4.** An OPLS-DA using the measured lipid levels (excluding PS, S1P, and dhS1P) and the clinical phenotypes was performed to investigate significant lipid modulations characteristic of COVID-19 subjects, compared with healthy subjects (A–F) or subjects with non-COVID-19 infectious diseases (G–L). The loading scatter plots (A, C, E, G, I, K) and important features of the T scores (B, D, F, H, J, L) for the OPLS-DA before symptom onset (A, B, G, H), on days 1–3 (C, D, I, J), and on days 4–6 (E, F, K, L) are shown.

**Supplemental Figure S5. OPLS-DA** **to investigate significant lipid modulations characteristic of COVID-19 subjects on days 7–9, days 10–12, and days 13–15.**

**Supplemental Figure S5.** An OPLS-DA using the measured lipid levels (excluding PS, S1P, and dhS1P) and the clinical phenotypes was performed to investigate significant lipid modulations characteristic of COVID-19 subjects, compared with healthy subjects (A–F) or subjects with non-COVID-19 infectious diseases (G–L). The loading scatter plots (A, C, E, G, I, K) and important features of the T scores (B, D, F, H, J, L) for the OPLS-DA on days 7–9 (A, B, G, H), on days 10–12 (C, D, I, J), and on days 13–15 (E, F, K, L) are shown.

**Supplemental Figure S6. OPLS-DA to investigate significant lipid modulations characteristic of COVID-19 subjects on days 16–18, days 19–24, and days 25–40.**

**Supplemental Figure S6.** An OPLS-DA using the measured lipid levels (excluding PS, S1P, and dhS1P) and the clinical phenotypes was performed to investigate significant lipid modulations characteristic of COVID-19 subjects, compared with healthy subjects (A–F) or subjects with non-COVID-19 infectious diseases (G–L). The loading scatter plots (A, C, E, G, I, K) and important features of the T scores (B, D, F, H, J, L) for the OPLS-DA on days 16–18 (A, B, G, H), on days 19–24 (C, D, I, J), and on days 25–40 (E, F, K, L) are shown.

**Supplemental Figure S7. PCA to identify significant lipids capable of differentiating COVID-19 from subjects with non-COVID-19 infectious diseases.**

A PCA using the measured lipid levels (excluding PS, S1P, and dhS1P) and the clinical phenotypes was performed to identify significant lipids capable of differentiating COVID-19 subjects from subjects with non-COVID-19 infectious diseases. The loading scatter plots are shown.

**Supplemental Figure S8. Time courses of the representative lipids in Figure 5.**

**Supplemental Figure S8.** Time courses of the representative lipids in Figure 5 are shown in a manner similar to Figure 1 and 2.

**Supplemental Figure S9. The time course of the representative lipids in Figure 6A, B.**

**Supplemental Figure S9.** The time course of the representative lipids in Figure 6A, B were shown in the similar manner to Figure 1 and 2.

**Supplemental Figure S10. The time course of the representative lipids in Figure 6C, D.**

**Supplemental Figure S10.** The time course of the representative lipids in Figure 6C, D were shown in the similar manner to Figure 1 and 2.

**Supplemental Figure S11. The ROC analyses for the prediction of the maximum severity at day 1 – 3 and day 4 – 6.**

**Supplemental Figure S11.** The area under curve (AUC) of the ROC analyses performed to discriminate the maximum severity 2~ group from the maximum severity 1 group (A, D), the maximum severity 3~ group from the maximum severity 1 or 2 (B, E), or the maximum severity 4 from others (C, F) at day 1 – 3 (A – C) and day 4 – 6 (D – F) are shown.

**Supplemental Figure S12. The ROC analyses for the prediction of the maximum severity at day 7 – 9 and day 10 – 12.**

**Supplemental Figure S12.** The area under curves (AUCs) of the ROC analyses performed to discriminate the maximum severity 2~ group from the maximum severity 1 group (A, D), the maximum severity 3~ group from the maximum severity 1 or and 2 (B, E), and maximum severity group 4 from maximum severity groups 1–3 (C, F) on days 7–9 (A–C) and days 10–12 (D–F).

**Supplemental Figure S13. ROC analyses for the prediction of maximum severity on days 13–15.**

**Supplemental Figure S13.** Area under curve (AUC) of the ROC analyses for differentiating maximum severity groups 2–4 from maximum severity group 1 (A), maximum severity groups 3 and 4 from maximum severity groups 1 and 2 (B), and maximum severity group 4 from maximum severity groups 1–3 (C) on days 13–15.

**Supplemental Figure S14. Modulations of sphingolipids and glycerophospholipids in subjects** **with bacterial pneumonia.**

**Supplemental Figure S14.** OPLS-DA using measured lipid levels to identify significant lipids capable of differentiating subjects with bacterial pneumonia (n = 80) from others (n = 29) among subjects with non-COVID-19 infectious diseases. A loading scatter plot (A) and important features of the T scores (B) for the OPLS-DA are shown.

**Supplemental Figure S15. Modulations of plasma lipids in mice treated with lipopolysaccharides, tissue factors, or histone.**

**Supplemental Figure S15.** Modulations of plasma sphingolipids and glycerophospholipids in mice treated with lipopolysaccharides (A, B, n = 6–7), tissue factor (C, D, n = 6–7), or histone (E, F, n = 6), prepared as described in the Methods section. (A, C, E) Volcano plots. (B, D, F) Important features of the T scores for the OPLS-DA.

**Supplemental Figure S16. Scheme showing the modulations of sphingolipids and glycerophospholipids in COVID-19.**

**Supplemental Figure S16.** Scheme summarizing the dynamic modulations of sphingolipids and glycerophospholipids in COVID-19.

**Supplemental Figure S17. Paired comparison of the lipid levels between specific time points and day 25–40 in a specific maximum severity group.**

**Supplemental Figure S17.** The difference in the lipid levels between specific time points and day 25–40 was longitudinally evaluated with the paired Wilcoxon signed-rank test in a specific maximum severity group. The average levels of the lipids in specific time points are shown as the ratio to day 25–40 (%). The blue, green, yellow, red, and black bars represent maximum severity 1 group, maximum severity 2 group, maximum severity 3 group, maximum severity 4 group, and all the patients, respectively.

**Supplemental Figure S18. Paired comparison of the lipid levels between specific time points and day 25–40 in a specific maximum severity group.**

**Supplemental Figure S18.** The differences in the lipid levels between specific time points and day 25–40 in a specific maximum severity group, evaluated with the paired Wilcoxon signed-rank test, are shown in the similar manner to Supplemental Figure S15.

**Supplemental Figure S19. Paired comparison of the lipid levels between specific time points and day 25–40 in a specific maximum severity group.**

**Supplemental Figure S19.** The differences in the lipid levels between specific time points and day 25–40 in a specific maximum severity group, evaluated with the paired Wilcoxon signed-rank test, are shown in the similar manner to Supplemental Figure S15.

**Supplemental Figure S20. Paired comparison of the lipid levels between specific time points and day 25–40 in a specific maximum severity group.**

**Supplemental Figure S20.** The differences in the lipid levels between specific time points and day 25–40 in a specific maximum severity group, evaluated with the paired Wilcoxon signed-rank test, are shown in the similar manner to Supplemental Figure S15.

**Supplemental Figure S21. Time courses for CRP and D-dimer.**

**Supplemental Figure S21.** The time courses of CRP (A–C) and D-dimer (D–F) are shown as violin plots. **p* < .05, ***p* < .01, ****p* < .001, *****p* < .0001.
